# Non-coding RNAs in necroptosis, pyroptosis and ferroptosis in cancer metastasis

**DOI:** 10.1038/s41420-021-00596-9

**Published:** 2021-08-11

**Authors:** Yan Liu, Qiuyun Chen, Yanan Zhu, Tiying Wang, Lijuan Ye, Lei Han, Zhihong Yao, Zuozhang Yang

**Affiliations:** 1 Bone and Soft Tissue Tumors Research Center of Yunnan Province, Department of Orthopaedics, The Third Affiliated Hospital of Kunming Medical University (Cancer Hospital of Yunnan Province), Kunming, Yunnan China; 2 Department of Pathology, The Third Affiliated Hospital of Kunming Medical University (Cancer Hospital of Yunnan Province), Kunming, Yunnan China

**Keywords:** Cancer, Mechanisms of disease

## Abstract

Distant metastasis is the main cause of death for cancer patients. Recently, the newly discovered programmed cell death includes necroptosis, pyroptosis, and ferroptosis, which possesses an important role in the process of tumor metastasis. At the same time, it is widely reported that non-coding RNA precisely regulates programmed death and tumor metastasis. In the present review, we summarize the function and role of necroptosis, pyrolysis, and ferroptosis involving in cancer metastasis, as well as the regulatory factors, including non-coding RNAs, of necroptosis, pyroptosis, and ferroptosis in the process of tumor metastasis.

## Facts

1. Programmed cell death has included apoptosis, autophagy-related cell death, necroptosis, ferroptosis, and pyroptosis.
2. Induction of programmed death of tumor cells exerts a vital role in the clinical treatment of cancer metastasis.
3. Non-coding RNA has participated in mediating multiple processes in tumor metastasis. At the same time, it has been found the non-coding RNAs have functions in regulating programmed death during cancer metastasis.
4. The interaction of necroptosis, pyroptosis, and ferroptosis is mediated by several key proteins such as NEK7, Tom20, caspase 1, etc.

## Open questions

1. How non-coding RNAs regulated the interactions between ferroptosis and necroptosis, ferroptosis, and pyroptosis?
2. The role of miRNAs, lncRNAs, or circRNAs regulated programmed cell death was not clearly clarified in the metastasis of cancers.
3. Is it a promising strategy in clinical cancer treatment to induce programmed cell death and identify the exact function of non-coding RNAs in the clinical therapy of cancers?

## Introduction

The metastasis of malignant tumors is the main reason for the failure of tumor therapy. Metastasis promotes cancer progression by degrading the extracellular matrix (ECM), mediating epithelial-to-mesenchymal transition (EMT), promoting tumor angiogenesis, etc. [1, 2]. This process is a complex molecular event involving multiple steps, multiple genes, and multiple cells [3]. Programmed cell death is an autonomous and orderly death. Apoptosis is often considered to be the traditional method of cell death. Now, programmed cell death has included apoptosis, autophagy, necroptosis, ferroptosis, and pyroptosis. Induction of programmed death of tumor cells exerts a vital role in the clinical treatment of cancer metastasis.

Metastatic tumor cells normally spread from the primary site, through lymphatics, blood vessels, or body cavities, and then colonize remotely, establish a local living environment, and continue to grow and infiltrate [4]. In recent years, non-coding RNA has participated in mediating multiple processes in tumor metastasis, such as epithelial–mesenchymal cell transformation and tumor angiogenesis [5]. Non-coding RNA mainly includes small nuclear RNA (snRNA), micro-RNA (miRNA), small interfering RNA (siRNA), piwi-interacting RNA (piRNA), small nucleolar RNA (snoRNA), circular RNAs, and lncRNAs [6]. Coding RNAs regulate gene expression during tumor development and metastasis through different pathways. At the same time, it has been found the non-coding RNAs have functions in regulating programmed death during cancer metastasis.

As we have summarized the regulatory mechanism of apoptosis and autophagy on tumor metastasis previously, the role of these two kinds of programmed death in tumor metastasis will not be repeated. This article mainly summarizes the role and regulatory mechanism of necroptosis, pyroptosis and ferroptosis in progression of tumor metastasis, and the regulation process of non-coding RNA on necroptosis, pyroptosis, and ferroptosis. At the same time, the relationship among necroptosis, pyroptosis and ferroptosis and some unresolved problems in the process of cancer metastasis was discussed, hoping to provide reference information for more in-depth theoretical and applied research in this field in the future.

## Necroptosis and cancer metastasis

### Necroptosis signaling pathway

Necroptosis is a caspases-independent cell death mode discovered recently [7]. The morphological characteristics of necroptosis cells are including incomplete cell membranes, crisis of intracellular energy metabolism, and release of inflammatory factors. Necroptosis is important in the development of various inflammatory diseases, neurodegenerative diseases, ischemic cardiovascular, cerebrovascular diseases, etc., as well as the metastasis of cancers. Necroptosis has been proved to have a dual effect in cancer: firstly, the key regulators of the necroptosis can promote the metastasis and progression of cancer alone or in combination; on the other hand, necroptosis can also play as a kind of “insurance”, it can prevent tumor development and metastasis when apoptosis is damaged. Considering the key function of necroptosis in the development of cancers, necroptosis is considered to be a new cancer treatment. It is reported that more and more drugs and compounds are inducing necroptosis to resist cancer. Studies have found that necroptosis is closely regulated by intracellular signal factors, such as the tumor necrosis receptor factor (TNFR) superfamily, pattern recognition receptors (PRRs), T cell receptors (TCRs), various chemotherapy drugs, etc. [8]. Among them, kinase receptor-interacting protein kinase (RIP) 1 and RIP 3 are important regulatory factors [9]. In addition, necroptosis can also be specifically inhibited by necrostatin-1 (Nec-1) [10].

First, necroptosis is initiated by the interaction of TNF and TNFR1, which induces the structural changes and formation of TNFR1 trimer, leading to the combination of RIP1, TNF receptor-related death domain (TRADD), cIAP1 (intracellular apoptosis). Apoptosis protein inhibitor 1) and cIAP2, TNF receptor-related factor 2 (TRAF2), and TRAF5 recruitment, this multimeric protein complex bound to the cell membrane is called complex I. In this complex, RIP1 is a key factor in cell regulation, which can be ubiquitinated by cIAP1/2, which in turn induces the activation of the classic NF-κB pathway and promotes cell survival. At the same time, RIP1 can also be deubiquitinated by CYLD, the NF-κB pathway is restricted, and the trend toward cell death pathway. Therefore, a cell death-inducing signal complex composed of RIP1, TRADD, caspase-8, and FAS-related death domain (FADD) is formed, called complex II, also called “nucleosome”, the formation of caspase-8 can induce the activation of caspase-8. Complex II involves the activation of the apoptotic and the necroptotic pathway. In complex II, activated caspase-8 cleaves RIP1 and RIP3, thereby inactivating them and moving to the apoptotic pathway, causing the cells to perform the apoptosis program [11]. When the activity of caspase-8 is inhibited, the shearing of RIP1 is blocked, necrosis of the key complex of pyroptosis is formed, and the cell death pathway directly shifts to necroptosis [12]. RIP1 is activated by phosphorylation at its N-terminus through serine residue 161. Activated RIP1 interacts with RIP3 to form a necrosome. Necrosome is an important key molecule in necroptosis. RIP3 activates its substrate MLKL, translocates to the cell membrane for necroptosis, changes the permeability of the cell membrane, and ultimately leads to cell death.

### Necroptosis in cancer metastasis

Necroptosis is important in the modulation of cancer metastasis, but the role of necroptosis is a dual role at different stages of cancer metastasis. In the early stage of distant colonization, the tumor cells spread via the circulatory system, in which tumor-cell-induced endothelial cell necroptosis promotes extravasation and metastasis [13] (Table 1). Another study showed that suppressing EMT was one effective way to inhibit invasion of radioresistant cancers, and inhibition of necroptosis such as depleting MLKL expression suppresses invasion of radioresistant nasopharyngeal carcinoma cells by suppressing EMT [14]. Moreover, knockdown aurora A-induced necroptosis which contributed to the increased survival times of mice with orthotopic KPC pancreatic ductal adenocarcinoma cancer (PDACs), and reduced tumor growth and metastasis [15]. Meanwhile, RIPK1 kinase inhibitors significantly repressed metastasis of both lung cancer cells and melanoma cells[16]. Additionally, it has been found RIP1 and MLKL are both positively associated with cancer parameters including N-cadherin, and suppressing necroptosis inhibited the metastasis of breast cancer [17]. Endothelial TGF-beta-activated kinase 1 (TAK1)-deficiency correlated with increased necroptosis and metastasis of endothelial cells. While others believe that the agents such as resibufogenin inhibit the occurrence and metastasis of colorectal cancer by RIP3-mediated necroptosis [18]. Additionally, sometimes, necroptosis of cancer cells possesses suppression in metastasis by activating anti-tumor immune responses to release damage-associated molecular patterns (DAMPs). While it also participates the adaptive immune suppression to promote tumor metastasis [19]. Thus, the role of necroptosis to regulate cancer metastasis appears to be controversial and highly dependent on the tumor stage.

**Table 1:** Noncoding RNAs regulates necroptosis in cancer metastasis.

Noncoding RNAs	Target gene	Function in human cancer metastasis
*LncRNA*		
SNHG20	miR-495	Overexpression of lncRNA SNHG12 regulates tumor metastasis by modulating HER2 via miR-495 [21]
HOTAIR	miR-331-3p	MiR-331-3p inhibited tumor metastasis by targeting MLLT10 in NSCLC [131]. Several lnc RNAs including UCA1 [132], MIAT [133] and XLOC_006390 [134] promoted cancer metastasis via regulating miR-331 in human cancers.
LncRNA3037	miR-15a	lncRNA3037 is down-regulated in tracheal tissue. Necroptosis was induced through lncRNA3037/miR-15a/BCL2-A20 signaling pathway [24]. miR-15a inhibits the growth and metastasis of human cancers by regulating Stat3 [135], Smad3 [136], Twist1 [137, 138], CXCL10 [139], etc.
LncRNA-107053293	miR-148a-3p	LncRNA-107053293 regulated necroptosis by acting as a ceRNA of miR-148a-3p [25]. miR-148a served as the tumor suppressor gene to inhibit cancer metastasis [140–142].
*miRNAs*		
miR-7-5p	SLC25A37 cTIMM50 NOVA2 HOXB13	miR-7 is reported to induce necroptosis by targeting SLC25A37 and TIMM50 to work as a tumor-suppressive gene [32]. MiR-7-5p was reported to inhibit tumor metastasis by targeting NOVA2 in NSCLC cancers [29], RELA in breast cancer stem cells [30], and HOXB13 in esophageal squamous cell carcinoma [31].
miR-141-3p	RIPK1 RIPK3 NF-kB NME	miR-141-3p suppressed upregulation of necroptosis-related molecules and interaction of receptor-interacting protein kinase 1 (RIPK1) and RIPK3 in LPS-treated Caco-2 cells by direct interacting with RIPK1 [36]. However, the role of miR-141-3p was inconsistent on the metastasis of various cancers [38, 40].
miR-425-5p	RIPK1	miR-425-5p was reported to negatively regulate the RIP1-mediated necroptosis by direct targeting the 3′UTR of RIP1 mRNA to decrease the expression of RIP1. Thus, miR-425-5p improved inflammation response and septic liver damage by inhibiting necroptosis [41].
miR-200a-5p	cRIPK3	Overexpression of miR-200a-5p induced RIP3-dependent necroptosis in vivo and in vitro [45].
miR-210	RIPK3	It promoted tumor metastasis by targeting E-cadherin in breast cancers [143]. It also promotes metastasis via NK-kB signing.
miR-223-3p	RIPK3	In acute kidney injury (AKI) models, miR-223-3p was obviously increased during 3-MCPD-dipalmitate-induced AKI, which inhibited RIPK3 expression by targeting the 3’ un-translated region of RIPK3 [47].
miR-500a-3p	MLKL	hsa-miR-500a-3P was obviously decreased in cisplatin-treated human tubular epithelial (HK2) cells, which significantly alleviated kidney injury by regulating MLKL-mediated necroptosis [48].
miR-210	HIF-3α E-cadherin	HIF-1alpha promoted necroptosis of macrophages by upregulating miR-210 in lesional macrophages [49]. Overexpression of miR-210 promoted cancer metastasis of breast cancer [143], bladder cancer [144], renal cell carcinoma [145], hepatocellular carcinoma [146] and colorectal cancer [147].
miR-181-5p	MMP-14	Atrazine induced necroptosis by regulating miR-181-5p and upregulating inflammation and glycometabolism in carp lymphocytes [50]. MiR-181 suppressed metastasis of human cancers via MMP-14 [148]. It also was used as putative biomarker form via lymph-node metastasis [149].
miR-16-5p	PI3K YAP1 Smad3 Twist 1 FGFR1	LPS-induced necroptosis was involved in miR-16-5p-PI3K/AKT signal in chicken tracheal epithelial cells [51]. miR-16-5p was reported to inhibit metastasis of chordoma cells, of bladder cancer via FGFR1. It also inhibited chordoma metastasis by targeging Smad3 [150].

### ncRNAs regulate necroptosis

**Fig. 1:**
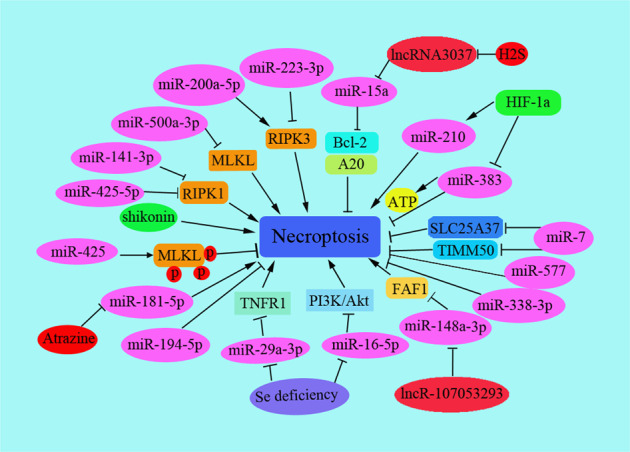
Necroptosis is regulated by ncRNAs. Necroptosis is regulated by noncoding RNAs including lncRNAs, miRNAs, and several important target proteins and signaling pathways are shown.

Recently, induction of necroptosis is thought to be an effective way to eliminate apoptosis-resistant cancer cells. Some compounds or natural products induce necroptosis and also inhibit the invasiveness of osteosarcoma cells [20]. Here, we mainly summarized the regulation process of non-coding RNA on necroptosis, hoping to provide a reference for researchers in cancer metastasis (Fig. 1).

### lncRNA

Compared with normal tissues of the same source, the expression of IncRNAs in a large number of tumor cells has changed. Recently, IncRNAs are reported to involve in tumor EMT and metastasis. Different IncRNAs regulate individual gene expression programs through epigenetic regulation or changes in transcription mechanisms. For example, in breast cancer cells, miR-495 was the target of SNHG20, and HER2 was regulated by SNHG20 via miR-495 to increase the ability of invasion and migration of tumor cells [21]. HER2 levels were also regulated by lncRNA HOTAIR by targeting at miR-331-3p in gastric cancer [22]. As necroptosis has totally different role in various tumor progression and metastasis [23], which functions as pro- or anti-tumoral role in cancer metastasis. However, there are only a few reports on the lncRNA in regulating cancer metastasis. Till now, several IncRNAs were reported to regulate necroptosis by function as competitive RNAs to increase or decrease the expression of target genes by interacting with miRNA. For instance, after treatment with hydrogen sulfide, necroptosis was induced and accompanied by decreased levels of lncRNA3037 and up-regulation of miR-15a in tracheal tissue. BCL2 and A20 were indirectly regulated by lncRNA3037, which directly bound to miR-15a and negatively regulate the level of miR-15a in the broiler trachea [24]. Moreover, lncRNA-107053293 regulated chicken tracheal cell necroptosis by function as a ceRNA of miR-148a-3p [25]. It was reported that decreased levels of miR-148a-3p suppressed the cell death of osteosarcoma [26]. Additionally, Fas-associated factor 1 (FAF1) was reported to activate the cell death machinery in the cytosol in Parkinson’s disease (PD) [27, 28].

### miRNA

Mounts of miRNAs regulate necroptosis in disease development and cancer metastasis. miR-7-5p (miR-7) is reported to possess the antitumor role. MiR-7-5p was reported to inhibit tumor metastasis by targeting NOVA2 in NSCLC cancers [29], RELA in breast cancer stem cells [30] and HOXB13 in esophageal squamous cell carcinoma [31]. In rhabdomyosarcoma (RMS), miR-7 showed the anti-tumor effect to induce necroptosis by targeting mitochondrial proteins SLC25A37 and TIMM50 [32]. SLC25A37 was significantly over-expressed in high cytotoxicity patients [33] and loss of TIM50 suppressed tumor cell growth and induced apoptosis in breast cancer [34] and loss of TIM50 suppressed tumor cell growth and induced apoptosis in breast cancer [35]. Several miRNAs were reported to regulate necroptosis by targeting and regulating RIP-1. For example, the effects of miR-141-3p were explored on intestinal epithelial cells, as well as the underlying molecular mechanism. The results showed that miR-141-3p suppressed upregulation of necroptosis-related molecules and interaction of receptor-interacting protein kinase 1 (RIPK1) and RIPK3 in LPS-treated Caco-2 cells by direct interacting with RIPK1 [36]. However, the role of miR-141-3p was inconsistent on the metastasis of different cancers. miR-141-3p promoted metastasis by targeting NME1in nasopharyngeal carcinoma [37], and also regulated osteoblast activity to increase the metastasis of prostate cancer [38]. While other researchers found that miR-141-3p suppressed metastasis of papillary thyroid cancer by targeting Yin Yang 1 [39] and the decreased miR-141-3p-promoted metastasis of prostate cancer by activating NF-kB pathway [40].

miR-425-5p was reported to negatively regulate the RIP1-mediated necroptosis by direct targeting the 3′UTR of RIP1 mRNA to decrease the expression of RIP1. Thus, miR-425-5p improved inflammation response and septic liver injury by down-regulating the occurrence of necroptosis [41]. Moreover, the cellular mechanism Parkinson’s disease was related to necroptosis promotion by miR-425 deficiency, for miR-425 targeted RIPK1 transcripts and promoted the MLKL phosphorylation [42].

RIP1 and RIP3 are necessary for necroptosis, and the complex regulates death receptor-induced necroptosis [43]. Especially, knockdown of RIP1 increases RIP3-mediated necroptosis at a special circumstance [44]. Several miRNAs were reported to regulate necroptosis by interacting and regulating RIP3. For instance, high levels of miR-200a-5p triggered RIP3-dependent necroptosis in vivo and in vitro [45]. In chemoresistant tumors, co-treatment with Kras-derived exosomes and carboplatin induced RIP3/TNFa-mediated necroptosis accompanied by miR-146/miR-210 modulation in metastatic lung cancer patients [46]. In acute kidney injury (AKI) models, miR-223-3p was obviously increased during 3-MCPD-dipalmitate-induced AKI, which inhibited RIPK3 expression by targeting the 3′ un-translated region of RIPK3 [47]. Furthermore, several other miRNAs were also reported to regulate necroptosis by regulating the different targets in the necroptosis signaling pathway. According to the analysis results by TargetScan software, several miRNAs had MLKL biding sites, including miR-194-5P, miR-338-3P, miR-500a-3P, and miR-577. The expression of these miRNAs is decreased in AKI, but only has-miR-500a-3P was obviously decreased in cisplatin-treated human tubular epithelial (HK2) cells, which significantly alleviated kidney injury by regulating MLKL-mediated necroptosis[48]. In hypoxic and cancer cells, HIF-1alpha (Hypoxia-Inducible Factor-1alpha) promoted necroptosis of macrophages by upregulating miR-210 and downregulating miR-383 levels in lesional macrophages and inflammatory bone marrow-derived macrophages. Among them, miR-210 was due to targeting 2,4-dienoyl-CoA reductase and contributed to the beta-oxidation of unsaturated fatty acids. miR-383 targeted with poly(ADP-ribose)-glycohydrolase (Parg) and affected the DNA damage repair pathway in bone marrow-derived macrophages and increased cell survival [49]. Atrazine promoted necroptosis by negatively regulating miR-181-5p and upregulating inflammation and glycometabolism in carp lymphocytes [50]. Additionally, Se deficiency regulated the miR-16-5p-PI3K/AKT pathway and exacerbated LPS-induced necroptosis in chicken tracheal epithelial cells by activating necroptosis-related genes [51].

## Pyroptosis in cancer metastasis

### Pyroptosis signaling pathway

Pyroptosis is a newly discovered form of programmed cell death characterized by a pro-inflammatory response, and it has both the characteristics of apoptosis and necrosis in morphology [52]. Pyroptosis mainly mediates the activation of a variety of Caspases including Caspase-1 through inflammasomes, causing shearing and multimerization of a variety of Gasdermin family members including GSDMD, causing cell proliferation and cell death [53, 54]. The inflammatory reaction occurs, accompanied by the release of contents and interleukins. The release of interleukin 1-β (IL-1β) and interleukin 18 (IL-18) can further expand the inflammatory response and recruit inflammatory cells [55]. At present, there are two main pyrolysis pathways that have been discovered, namely the classic pyrolysis pathway and the non-classical pyrolysis pathway. The former is mediated by caspase1-dependent inflammasomes, and the latter is mediated by caspase 4, 5, 11, and lipopolysaccharide (LPS) [56]. In recent years, researchers have discovered that pyrolysis is closely related to a variety of human diseases, such as infectious diseases, cardiovascular and cerebrovascular diseases, immune system defects, and tumors [57, 58]. In the study of tumor pathogenesis, researchers found that pyrolysis can affect tumor cell proliferation, migration and invasion ability.

**Fig. 2:**
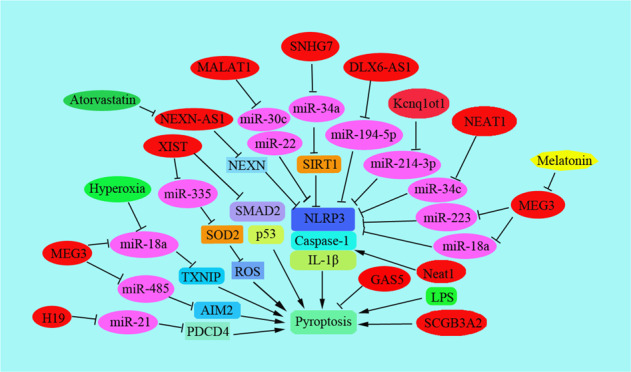
Pyroptosis is regulated by ncRNAs. Pyroptosis is regulated by noncoding RNAs including lncRNAs and miRNAs. The related key targeting proteins are shown, such as NLRP3, caspase-1, and IL-1beta.

Pyroptosis is a double-edged sword for tumor progression and metastasis [58] (Table 2). Firstly, pyrolysis can inhibit the development of the tumors as an innate immune mechanism. For example, alpinumisoflavone (AIF) inhibited hepatocellular carcinoma (HCC) cell metastasis by promoting NLRP3 inflammasome-mediated pyroptosis, suggesting pyroptosis inhibited cell metastasis in AIF-treated HCC cells [59]. FL118 activated NLRP3-ASC-Caspase-1-mediated pyroptosis, which suppressed the metastasis of colorectal cancer cells [60]. Moreover, resibufogenin triggers caspase-1-dependent pyroptosis through ROS-mediated NF-kappaB suppression to inhibit metastasis of non-small cell lung cancer [61]. Secondly, pyroptosis provides a suitable microenvironment for tumor growth to exert the role of pro-inflammatory cell death. The key components of the pyrolysis pathway: inflammasome, gasdermin protein, and pro-inflammatory cytokines are all related to tumor occurrence and metastasis. For instance, in the early steps of metastasis, inflammation response recruited distant MDSCs to induce metastasis of breast cancer partly by pyroptosis-induced IL-1beta generation and downstream CCL2, CCL5, and CXCL5-related signal pathway [62]. The other paper reported that the higher expression of pyroptosis signaling pathway effectors caspase-1, IL-1beta, and GSDMD was negatively related to tumor size and lymph node metastasis [63]. The levels of pyroptosis signaling pathway effectors caspase-1, IL-1beta, and GSDMD involve in the invasion and metastasis of breast cancer. By testing 108 cases of breast cancer tissues and 23 cases of para-cancerous benign tissues, the results showed the higher expression level of caspase-1, IL-1beta, and GSDMD were associated with the lower histopathologic grade of breast cancer, the smaller of tumor size, the lower possibility of metastasis, and the better the prognosis of breast cancer [63]. Thus, it is important to clear out the regulatory mechanism of pyroptosis in the process of tumor metastasis, which has important reference significance for the therapy of cancer (Fig. 2).

**Table 2:** Noncoding RNAs regulates pyroptosis in cancer metastasis.

LncRNAs	Target gene	Function in human cancer metastasis
SNHG7	miR-34a	Interference with SNHG7 decreased the levels of SIRT1 via regulating the expression of miR-34a and promoted pyroptosis in liver cancer patients [64]. miR-34a suppressed metastasis of human cancers by targeting specific genes, including YY1 in liver cancer [151], CCL22 in renal cell carcinoma [152], CD44 in osteosarcoma cells [153], and prostate cancers [154].
Kcnq1ot1	miR-214-3p miR-486a-3p	LncRNA Kcnq1ot1 induced pyroptosis in diabetic corneal endothelial kerotopathy [155]. Kcnq1ot1 induced pyroptosis was due to inhibiting miR-486a-3p and upregulating NLRP3 [156]. Knockdown Kcnq1ot1 inhibited gasdermin D cleavage to regulate pyroptosis [65].
lncRNA GAS5	miR-34b-3p miR-452-5p	LncRNA GAS5 was associated with the progression of ovarian cancer by regulating the formation of inflammasome and pyroptosis [67]. lncRNA GAS5/miR-452-5p downregulated oxidative stress and pyroptosis [157]. Moreover, GAS5 inhibited pyroptosis in diabetic cardiomyopathy by targeting miR-34b-3p/AHR [158].
LncRNA MEG3	miR-485 miR-223 miR-184 miR-21	Lnc MEG3 promoted pyroptosis by down-regulating the levels of miR-485 and up-regulating the levels AIM2 [68]. Additionally, melatonin inhibited pyroptosis by regulating the miR-223/NLRP3 pathway [89]. MEG3 inhibited metastasis via targeting miR184 in myeloid leukemia [159]. In gastric cancer, MEG3 inhibited metastasis by regulating miR-21 [70].
LncRNA XIST	miR-335 miR-137 miR-139-5p miR-217	Interference with XIST inhibited NSCLC development by activating miR-335/SOD2/ROS pathway mediated pyroptosis [77]. XIST promoted metastasis of glioma by miR-133a/SOX4 [73]. It also promoted metastasis of colorectal cancer by regulating the miR-137-EZH2 pathway [74]. And XIST promoted metastasis of bladder cancers via miR-139-5p-mediated Wnt/β-catenin pathway [160] and induced metastasis of melanoma by sponging miR-217 [76]. Silencing XIST promoted pyroptosis and suppressed NSCLC development by inducing ROS production and activating NLRP3 [77].
LncRNA Neat1	miR-34c miR-146b-5p miR-224-5p miR-382-3p	The lncRNA Neat1 stabilized the mature caspase-1 to promote pyroptosis [78]. Neat1 promoted metastasis in human various cancers, by inhibiting miR-146b-5p [161] in breast cancers, targeting miR-224-5p in malignant melanoma [162], by mediating miR-382-3p in ovarian cancer [163].
lncRNA MALAT1	miR-22 miR-23c	lncRNA MALAT1 promoted pyroptosis as the ceRNA to competitively bind miR-22, which led to the levels of NLRP3 was affected. This might be a new way in the clinical therapy for atherosclerosis [81, 164].
lncRNA DLX6-AS1	miR-223-3p miR-641 miR-577	In AKI patients, higher levels of DLX6-AS1 were observed. Silencing DLX6-AS1 suppressed the pyroptosis of HK-2 cell through miR-223-3p/NLRP3 signaling in LPS-induced acute kidney injury [82]. DLX6-AS1 promoted metastasis in prostate cancer via mediating LARGE methylation [165]. DLX6-AS1 promoted metastasis via miR-641/HOXA9 pathway in osteosarcoma [166] and targeting miR-577 in esophageal squamous cell carcinoma [167]. Inhibition of DLX6-AS1 suppressed metastasis via Notch signaling in human epithelial ovarian cancers [168].
lncRNA H19	miR-21 miR-675-5p miR-138 miR-29b-3p miR-6515-3p	lncRNA-H19 functioned as the sponge of miR-21 to stimulate PDCD4 expression and formed a ceRNA in ischemic cascade [83]. H19 promoted tumor metastasis by targeting miR-675-5p [169], miR-138 [170], miR-29b-3p [171], miR-6515-3p [172].

### lncRNAs regulate pyroptosis in cancer metastasis

In liver cancers, SNHG7 worked as a ceRNA of miR-34a, and SIRT1 was proved to be a direct target of miR-34a. Interference with SNHG7 down-regulated the expression of SIRT1, but up-regulated the levels of NLRP3, caspase-1, and interleukin-1beta, which induced pyroptosis, suggesting NLRP3-dependent pyroptosis was induced through SNHG7/miR-34a/SIRT1 signaling pathway during liver cancer [64]. Kcnq1ot1 regulated the level of caspase-1 by working as a sponge of miR-214-3p. Interference with the level of Kcnq1ot1 suppressed gasdermin D cleavage and the secretion of IL-1beta to promote pyroptosis in high glucose-treated cardiac fibroblasts [65]. Deletion of Kcnq1ot1 inhibited pyroptosis by regulating miR-214-3p and caspase-1 in diabetic cardiomyopathy [66]. Long non-coding RNA growth arrest-specific transcript 5 (lncRNA GAS5) was obviously down-regulated in ovarian cancer tissues, which was proved to be associated with inflammasome formation and pyroptosis [67]. Lnc MEG3 promoted pyroptosis and induced inflammation, which down-regulated the levels of miR-485 and up-regulated the levels of AIM2 inflammasome [68]. It has been reported MEG3 inhibited tumor metastasis by regulating the Wnt/beta-catenin pathway in oral squamous cell carcinoma [69], regulating the expression of miR-21 in gastric cancer (GC) [70], and miR-183 expression in human pancreatic cancers [71]. However, MEG3 promoted metastasis of melanoma via modulating the miR21/E-cadherin pathway [72].

LncRNA X inactive-specific transcript (XIST) promoted metastasis of glioma by miR-133a/SOX4 [73]. LncRNA XIST promoted metastasis of glioma by miR-133a/SOX4 [74]. And XIST promoted metastasis of bladder cancers via miR-139-5p-mediated Wnt/β-catenin pathway [75] and induced metastasis of melanoma by sponging miR-217 [76]. Silencing XIST promoted pyroptosis and suppressed NSCLC development by stimulating ROS production and activating the NLRP3 inflammasome. Moreover, inhibiting the expression of XIST inhibited NSCLC progression by activating miR-335/SOD2/ROS pathway mediated pyroptosis [77]. The lncRNA Neat1 induced the stabilization of mature caspase-1 leading to the higher secretion of interleukin-1β and activation of inflammasomes in macrophages, suggesting lncRNA Neat1 promoted the pyroptosis [78]. The level of XIST was decreased in several types of tumors. Silencing of lncRNA XIST suppressed the cell proliferation of NSCLC and promoted chemosensitivity to cisplatin partly by its binding to the TGF-beta effector SMAD2 and stimulating pyroptosis [79]. Intracellular LPS-induced pyroptosis of innate immune cells. Secretoglobin (SCGB)3A2, triggered pyroptosis of the RAW264.7 cells and inhibited the tumor cell growth in vitro. The results showed that LPS triggered pyroptosis of the immune cells, which clarified the direct effects of LPS on tumor cells [80].

For the endothelial cells under high-glucose stress, lncRNA MALAT1 promoted pyroptosis as the ceRNA to competitively bind miR-22, which led to the levels of NLRP3 was affected. This might be a new way in the clinical therapy for atherosclerosis (AS) [81]. The DLX6-AS1 expression increased in AKI patients, and DLX6-AS1 worked as the sponge of miR-223-3p, which led to the repressing expression of miR-223-3p in HK-2 cells. Silencing DLX6-AS1 suppressed the pyroptosis of HK-2 cells through miR-223-3p/NLRP3 signaling in LPS-induced AKI [82]. Ischemia-reperfusion (I/R) promoted lncRNA-H19 expression, which led to the significant imbalance of NLRP3/6 inflammasome, as well as the high level of cytokines and microglial pyroptosis. Actually, lncRNA-H19 functioned as the sponge of miR-21 to stimulate PDCD4 expression and formed a ceRNA in an ischemic cascade [83].

Upregulation of lncRNA NEAT1 was accompanied by the increased level of pyroptosis in the diabetic nephropathy (DN) model. Cell pyroptosis was regulated by NEAT1 and miR-34c, the target gene of NEAT1, by mediating NLRP3 in DN, caspase-1, and IL-1β [84]. Decreased expression of lncRNA MALAT1 inhibited renal tubular epithelial pyroptosis by upregulating miR-23c and down-regulating the levels of ELAVL1, NLRP3, Caspase-1, and the IL-1beta in DN [85]. LncRNA MALAT1 promoted the pyroptosis of HK-2 cells by suppressing the interaction between miR-30c and its target gene NLRP3 [86]. Atorvastatin upregulated NEXN-AS1 and the levels of NEXN, which suppressed pyroptosis by down-regulating the endogenous levels of the canonical inflammatory biomarkers NLRP3, caspase-1, GSDMD, IL-1beta, and IL-18. Thus, atorvastatin inhibited pyroptosis via NEXN-AS1/NEXN pathway in vascular endothelial cells [87].

Silencing KCNQ1OT1 obviously suppressed H2O2-induced SRA01/04 cell pyroptosis, which led to increased expression of miR-214 and down-regulated the level of caspase-1. Thus, pyroptosis is regulated by the KCNQ1OT1-miR-214-caspase-1 pathway [88]. Melatonin inhibited the pyroptosis of endothelial cells by regulating the lncRNA MEG3/miR-223/NLRP3 pathway during AS process [89]. Here, MEG3 worked as a sponge by complementarily suppressing the role of miR-223, increasing the NLRP3 level, and enhancing the pyroptosis of endothelial cells. The expression of MEG3 was promoted in lung tissues under the condition of hyperoxia. Silencing MEG3 inhibited pyroptosis to alleviate hyperoxia lung injury by suppressing NLRP3 inflammasome and caspase-1-related signaling via regulating miR-18a-thioredoxin-interacting protein (TXNIP) signaling [90].

### Ferroptosis

In recent years, the influence of free radicals on the body and cytotoxicity have received more and more attention. Ferroptosis is a newly discovered iron-dependent programmed cell death (PCD) [91]. Its morphological characteristics are shrinkage of cell volume and increase of mitochondrial membrane density. The main feature of this process is the accumulation of iron-dependent lethal lipid ROS rather than cell death in the form of apoptosis [92]. Specifically, ferroptosis is due to the failure of the membrane lipid repair enzyme-glutathione peroxidase (GPX4), resulting in the accumulation of reactive oxygen radicals (ROS) on membrane lipids, and this accumulation process requires the participation of ions [93]. It regulates cell death in many diseases such as tissue I/R injury, acute renal failure, neurodegenerative disease, and cancer progression.

The main mechanism of ferroptosis is that under the action of divalent iron or ester oxygenase, it catalyzes the unsaturated fatty acids highly expressed on the cell membrane to cause liposome peroxidation, thereby inducing cell death; in addition, it is also manifested in the antioxidant system (Glutathione GSH and GPX4) expression is decreased [94]. The ferroptosis pathway was including the induction of ferroptosis by inhibiting the cystine-glutamate transport receptor (systemXC-), p53-mediated ferroptosis, and direct inhibition of GPX4-induced ferroptosis [95]. Although the signaling pathways are different, the upstream pathways ultimately affect the activity of glutathione peroxidase (GPXs) directly or indirectly, reducing the antioxidant capacity of cells and increasing the lipid peroxidation reaction. Lipid active oxygen increases, causing ferroptosis [96]. Ferroptosis inducers can be divided into two categories: the first category of inducers includes erastin, sulfasalazine, and sulfoximine, etc. This category of inducers inhibits system Xc- (glutamate and cystine The antiporter) function and reduces the content of glutathione (GSH) in the cell to induce cell redox imbalance; the second type of inducer includes RSL3, DPI7, DPI10, DPI12, DPI13, etc. Synthetic compounds, these inducers directly inhibit GPX4 and also cause the accumulation of peroxide in the cell. Ultimately, depending on the abnormal metabolism of iron ions in cells, the accumulation of reactive oxygen species (ROS) leads to ferroptosis [97].

**Fig. 3:**
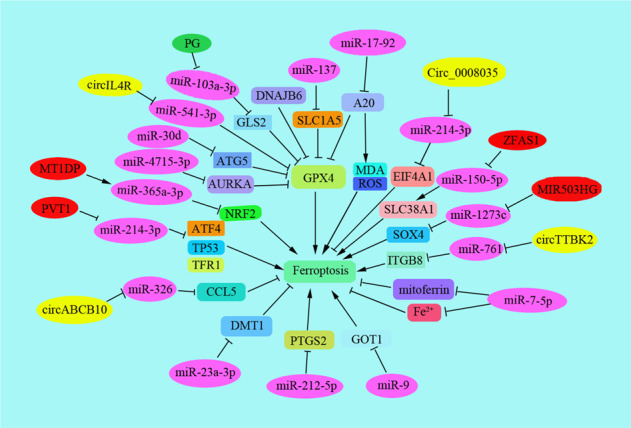
Ferroptosis is regulated by ncRNAs. Ferroptosis is regulated by noncoding RNAs including lncRNAs, circRNAs and miRNAs. The key targeting proteins are shown.

There are many genes and enzymes involved in the regulation of ferroptosis, including P53, GPX4, ACSL4, SCL7A11, and so on. In recent years, more and more non-coding RNAs have been shown to regulate ferroptosis in tumor cells, such as miR-9 and miR-137 (Table 3). Ferroptosis plays the opposite role either inhibition or promotion in tumor metastasis by regulating multiple signaling molecules in the tumor microenvironment [98]. It might be a promising strategy to induce ferroptosis to overcome chemotherapeutic drug resistance and inhibit cancer metastasis in clinical anti-cancer therapy. Therefore, this article summarizes the regulation process of non-coding RNAs on ferroptosis during tumor development and metastasis and provides references for further elucidating the mechanism of ferroptosis in tumor metastasis (Fig. 3).

**Table 3:** Noncoding RNAs regulates ferroptosis in cancer metastasis.

Noncoding RNAs	Target gene	Function in human cancer metastasis
*LncRNA*		
MIR503HG	miR-1273c [99] EMT-related proteins [173]	The expression of lncRNA MIR503HG was decreased in bladder cancer tissues, which was associated to lymph node metastasis. Overexpression of MIR503HG inhibited tumor metastasis by decreasing the EMT-related protein levels, such as ZEB1, Snail, N-cadherin, etc. [173]. In hepatocellular carcinoma, lncRNA miR503HG exerted a metastatic tumor suppression role by inhibiting NF-kB pathway via modulating HNRNPA2B1 ubiquitination [174]. Importantly, treatment with XAV939 decreased the expression of lncRNA MIR503HG by sponging miR-1273c and regulating SOX4 level. The XAV939 induced decreased expression of SLC7A11 suppressed NSCLC development through the ferroptosis pathway [99].
MT1DP	MDA and ROS [100] ADAM10-JAK-STAT [101]	Overexpression of lncRNA MT1DP promoted MDA production and ROS levels, which sensitized lung cancer cells to erastin-induced ferroptosis [100]. It has reported miR-365a-3p inhibited metastasis of colorectal cancer by regulating ADAM10-JAK-STAT pathway [101].
PVT1	miR-214 [102] miR-361-3p/SOX9 [175] miR-145 [176] miR-526b/FOXC2 [177] miR-484 [178] miR-106a-5p/HK2 [179] miR-125 [180] miR-186-5p [181]	Lnc RNA PVT1 promoted ferroptosis in vivo by down-regulating miR-214 and exsisted a positive feedback loop of lncRNA PVT1/miR-214/p53 [102]. PVT1 was reported to promoted metastasis of various human cancers such as lung cancer [175, 182], CRC [176], osteosarcoma [178], hepatocellular carcinoma [181], gastric cancer cells [180] and oral squamous cell carcinoma [179].
ZFAS1	miR-150-5p/SLC38A1 [103]	Interference with ZFAS1 inhibited ferroptosis by sponging miR-150-5p to down-regulate the expression of SLC38A1 [103]. Meanwhile, MiR150 was closely related to the metastasis of nasopharyngeal carcinoma [183].
Circ RNAs		
Circ_0008035	miR-599/EIF4A1 [104] miR599/c-Myc [184]	Circ_0008035 was increased in GC tissues to suppress ferroptosis via miR-599/EIF4A1 axis [104]. Additionally, miR599/c-Myc pathway was also involved in the metastasis of esophageal squamous cell carcinoma [184].
CircABCB10	miR-326/CCL5 [105] miR-326/SLC27A4 [185] miR-326/FSCN1 [186] miR-326/TWIST1[187]	Interference with circABCB10 suppressed the cell ferroptosis by regulating the miR-326/CCL5 axis in rectal cancer. CircABCB10 and CCL5 were upregulated but miR-326 was downregulated in rectal cancer [105]. Moreover, miR-326 promoted metastasis in gastric cancer [186], lung cancer [188], liver cancer [185], and endometrial cancer [187].
CircIL4R	miR-541-3p/GPX4 [106] miR-541-3p/TMPRSS4 [189]	circIL4R was abnormally overexpressed in HCC. CircIL4R knockdown impeded oncogenesis and expedited ferroptosis of HCC cells by the miR-541-3p/GPX4 network [106]. It has also reported miR-541-3p inhibited the migration of HCC cells via suppressing the level of TMPRSS4 [189].
Circ-TTBK2	miR-761/ ITGB8 [107] miR-761/ING4 and TIMP2 [190] miR-761/Ras [191]	circ-TTBK2 knockdown suppressed invasion, and promoted ferroptosis via targeting ITGB8 by sponging miR-761 in glioma[107]. MiR-761 promoted metastasis of NSCLC by targeting ING4 and TIMP2 [190].
*miRNAs*		
miR-202	PIK3CA [192]	lncRNA MALAT1 promoted osteosarcomas metastasis by sponging miR202 [193]. miR202 inhibited prostate cancer metastasis by targeting PIK3CA [192]
miR-103a-3p	GLS2 [194] KLF4 [195] LATS2 [196] DAPK and KLF4 [197]	miR-103a promoted metastasis of gastric cancer by targeting KLF4 [195], and promoted metastasis of hepatocellular carcinoma by inhibiting LATS2 [196]. Moreover, PG exerted anti-tumor role in gastric cancer (GC) by downregulating inhibitory effect of miR-103a-3p on glutaminase 2 (GLS2) expression, which was involved in ferroptosis during the progression of GC [194].
miR-214-3p	ATF4 [111]	MicroRNA-214-3p promoted ferroptosis in hepatoma cells partly by decreased the expression of ATF4, which obviously decreased the size and weight of xenografted tumors [111]
miR-137	SLC1A5 [112]	miR-137 negatively regulated ferroptosis by regulating SLC1A5 in melanoma. However, interference with miR-137 increased the antitumor effects of erastin by promoting ferroptosis, suggesting promotion of ferroptosis was a potential treatment for melanoma [112].
miR-17-92	A20-ACSL4 [113]	miRNA-17-92 is an oncogenic miRNA which is associated with lymph node metastasis in oesophageal adenocarcinoma [198]. But in gastric cancers, miRNA-17-92 was negatively associated with metastasis [199]. Overexpression of miR-17-92 suppressed the cell death of endothelial HUVEC cells and reduced ROS generation. Moreover, miR-17-92 suppressed the erastin-induced ferroptosis [113].
miR-4715-3p	AURKA [115] RAC1 [200]	The miR-4715-3p were methylated in upper gastrointestinal adenocarcinoma (UGC). Knockdown of miR-4715-3p in UGCs inhibited GPX4 induced cell death [115]. Interference with LCAT1 inhibited metastasis in the mouse xenografts mediated by miR-4715 [200].
miR-522	ALOX15 [116] DENND2D [201]	lipoxygenase 15 (ALOX15) was associated to lipid-ROS production in human gastric cancer, and exosome-miR-522 worked as a potential inhibitor of ALOX15 [116]. Interference with miR-522 inhibited metastasis of NSCLCs by directly targeting DENN/MADD domain containing 2D (DENND2D) [201].
miR-212-5p	Ptgs2 [117] Sirt2 [202] TCF7L2 [203]	miR-212-5p attenuated ferroptotic neuronal death by targeting Ptgs2 [117]. Additionally, miR-212-5p regulated cancer metastasis by targeting Sirt2 in colorectal cancer [202] and TCF7L2 [203] in human cervical cancer.
miR-23a-3p	DMT1 [118] CDH1 [204] TSGA10 [205] Sprouty2 [206]	HUCB-MSCs-exosomes inhibited DMT1, the target gene of miR-23a-3p to suppress ferroptosis and decrease myocardial injury [118]. miR-23a regulated metastasis of various human cancers with different mechanism. For instance, interference with miR-23a facilitated metastasis of cutaneous melanoma [207], but it also promoted mammary carcinoma cell metastasis by targeting Sprouty2 [206].
miR-30d	FTH1 and GPX4 [119] SOX9 [208]	Interference with miR-30d increased the expression of FTH1 and GPX4 in H9C2 cells to promote ferroptosis [119]. miR-30d was reported to involved in regulation the metastasis of retinoblastoma cells via miR-30d/SOX9/ZEB2 [208].

### LncRNAs regulate ferroptosis in cancer metastasis

Recently, lncRNAs play an important role to regulate ferroptosis by targeting miRNAs in cancer progression. For example, treatment with XAV939 decreased the expression of lncRNA MIR503HG, which obviously suppressed NSCLC progression by sponging miR-1273c and regulating SOX4 level. The XAV939 decreased the expression of SLC7A11, which suppressed NSCLC progression through the ferroptosis pathway [99]. Overexpression of lncRNA metallothionein 1D pseudogene (MT1DP) promoted malondialdehyde (MDA) production and ROS levels and sensitized A549 and H1299 cells to erastin-induced ferroptosis by regulating the level of NRF2 via stabilizing miR-365a-3p [100]. miR-365a-3p suppressed metastasis of colorectal cancer by regulating the ADAM10–JAK–STAT pathway [101]. Lnc RNA PVT1 promoted ferroptosis in vivo by down-regulating miR-214 and upregulating the expression of TFR1 and TP53. It could be exsisted a positive feedback loop of lncRNA PVT1/miR-214/p53 [102]. Silencing lncRNA ZFAS1 attenuated ferroptosis by sponging miR-150-5p to down-regulate the expression of SLC38A1 [103].

### Circ RNAs regulate ferroptosis in cancer progression

Circular RNAs works as a sponge of miRNAs to regulate cancer progression. Here, we summarized several circRNAs regulated ferroptosis and contributed to promote or inhibit cancer progression. Circular RNAs Circ_0008035, acting as a sponge of miR-599, Circ_0008035 was up-regulated in GC tissues, which contributes to tumorigenesis and represses apoptosis and ferroptosis in GC via miR-599/eukaryotic initiation factor 4A1 (EIF4A1) axis [104]. Interference with circABCB10 suppressed the cell ferroptosis by regulating the miR-326/C–C motif chemokine ligand 5 (CCL5) axis in rectal cancer [105]. CircABCB10 and CCL5 were upregulated but miR-326 was downregulated in rectal cancer. Circ-interleukin-4 receptor (circIL4R) was abnormally overexpressed in hepatocellular carcinoma (HCC). CircIL4R knockdown impeded oncogenesis and expedited ferroptosis of HCC cells by the miR-541-3p/GPX4 network [106]. Circular RNA Tau tubulin kinase 2 (circ-TTBK2) is a cancer-associated gene. circ-TTBK2 knockdown suppressed invasion, and promoted ferroptosis via targeting ITGB8 by sponging miR-761 in glioma [107].

### miRNAs regulate ferroptosis in cancer progression

Triggering ferroptosis is useful to inhibit cancer progression, such as inhibition of iron–sulfur cluster biosynthetic enzyme NFS1 inhibited lung adenocarcinoma by triggering ferroptosis [108]. Sometimes, ferroptosis contributed to the antitumour effects of the tumor suppressors such as p53 and BAP1. During this process, more and more miRNAs are reported to involve in ferroptosis during cancer metastasis. For example, in acute myeloid leukemia, differentially expressed GPXs were involved in cell proliferation, cancer progression, apoptosis, and cell cycle pathways involving cancer-related miRNAs (such as miR-202 and miR-181) [109]. Physcion 8-O-beta-glucopyranoside (PG) exerted an anti-tumor role in GC by downregulating inhibitory effect of miR-103a-3p on glutaminase 2 (GLS2) expression, and the upregulating ROS level, intracellular Fe(2+) level, and malondialdehyde (MDA) generation [110]. MicroRNA-214-3p promoted ferroptosis in hepatoma cells partly by decreased the expression of ATF4, which obviously decreased the size and weight of xenografted tumors [111]. miR-137 negatively regulates ferroptosis by directly targeting SLC1A5 in melanoma cells. However, interference with miR-137 increased the antitumor effects of erastin by promoting ferroptosis, suggesting promotion of ferroptosis was a potential treatment for melanoma [112]. miRNA-17-92 is an oncogenic miRNA that has a vital role in tumor development. Overexpression of miR-17-92 suppressed the cell death of endothelial HUVEC cells and reduced ROS generation. Moreover, miR-17-92 suppressed the erastin-induced ferroptosis by regulating the A20–ACSL4 pathway [113]. High expression of miR-9 down-regulated erastin- and RSL3-induced ferroptosis through regulating glutamic-oxaloacetic transaminase GOT1 in melanoma cells [114]. While, miR-4715-3p were methylated in upper gastrointestinal adenocarcinoma (UGC). Knockdown of miR-4715-3p in UGCs inhibited GPX4 and induced cell death [115]. Additionally, several miRNAs were reported to regulate ferroptosis, but how the miRNAs regulated ferroptosis to regulate cancer metastasis was not clearly reported. For instance, lipoxygenase 15 (ALOX15) was associated to lipid-ROS production in human GC, and exosome-miR-522 worked as a potential inhibitor of ALOX15 [116]. miR-212-5p attenuated ferroptosis neuronal death by targeting Ptgs2 [117]. HUCB-MSCs-exosomes inhibited DMT1, the target gene of miR-23a-3p to suppress ferroptosis and decrease myocardial injury [118]. HUCB-MSCs-exosomes inhibited DMT1, the target gene of miR-23a-3p to suppress ferroptosis and decrease myocardial injury [119]. While in rat models, down-regulating the expression of miR-30b-5p and coadministration with ferroptosis inhibitors decreased the preeclampsia (PE) symptoms [120]. Overexpression of miR-30b-5p in PE models had a key function in ferroptosis, by decreasing the expression of Cys2/glutamate antiporter, PAX3, and ferroportin 1 (an iron exporter), leading to decreased GSH and increased labile Fe(2+), which revealed miR-30b-5p is a potential therapeutic target for PE [120]. However, the role and regulation of miR-30 family-involved ferroptosis in cancer metastasis was not still clarified, which was an interesting question till now.

### The crosstalk between necroptosispyroptosis and ferroptosis

**Fig. 4:**
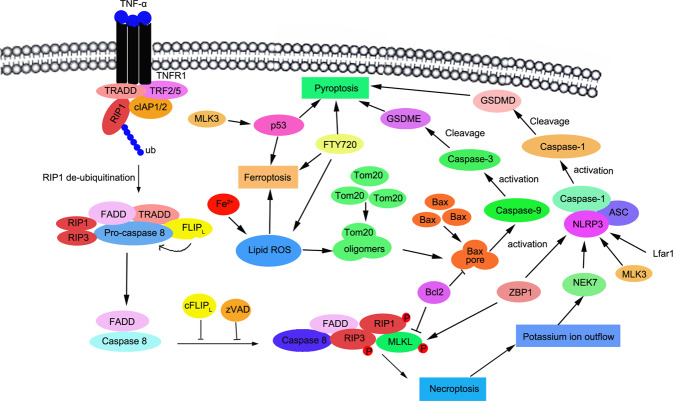
The crosstalk between necroptosis, pyroptosis, and ferroptosis. Several studies showed that there is an interaction between necroptosis, pyroptosis, and ferroptosis. RIP1 or RIP3-mediated necroptosis contributes to the membrane damage. Potassium ion outflow activates NEK7, which leads to the cleavage of GSDMD. Additionally, Iron activates ROS for GSDME-dependent pyroptosis in melanoma cells, suggesting there is a crosstalk between ferroptosis, pyroptosis and necroptosis.

Enormous studies discovered crosstalk between these programmed cell deaths. The interaction of necroptosis, pyroptosis, and ferroptosis is mediated by several key proteins such as NEK7, Tom20, caspase 1, etc. (Fig. 4). For example, necroptosis and pyroptosis is both able to induce lytic cell death. NEK7 interacted with NLRP3 to regulate pyroptosis [121]. Knockdown of lncRNA Lfar1 inhibited NLRP3 inflammasome-mediated pyroptosis in hepatic stellate cells [122]. ZBP1 works as the sensor of fungal infection to activate pyroptosis and necroptosis [123]. Bcl-2 is found to regulate pyroptosis and necroptosis by targeting BH3-like domains in GSDMD and MLKL [124]. Caspase-8 is an important protein to work as a switch for necroptosis and pyroptosis [125]. Moreover, iron is reported to induce oxidative stress by increasing ROS levels and regulate ferroptosis and necroptosis. Tom20 is oxidized by iron-activated ROS signaling and triggers pyroptosis of melanoma cells by inducing GSDME cleavages [126]. Mixed-lineage kinase 3 (MLK3) regulated pyroptosis through NF-kB/NLRP3 signaling and ferroptosis via JNK/p53 pathway during myocardial fibrosis [127]. Transcription Factor p53 prompted pyroptosis to suppress tumor growth in NSCLC patients [128, 129]. Ferroptosis is also induced which is dependent on P53 in liver fibrosis and effectively inhibit HSC activation [130]. This demonstrates p53 is a key factor to induce both pyroptosis and ferroptosis. Undoubtedly, enormous noncoding RNAs are involved in the regulation of the crosstalk between these programmed cell deaths. However, there are few reports on the regulation of noncoding RNAs in the crosstalk between necroptosis, ferroptosis, and pyroptosis.

### Conclusion and perspectives

Tumor metastasis is the main course of death from nearly all types of cancers. Recently, programmed cell death included several other types of cell death besides apoptosis and cell autophagy-induced cell death, such as necroptosis, ferroptosis, or pyroptosis, which has been reported to play different role in tumor progression. Induction of programmed cell death of tumors exerts a vital role in the clinical treatment of cancer metastasis. It is important to clearly elucidate the detailed regulatory mechanism of programmed cell death (PCD) during cancer development, in this review, we have summarized and discussed how non-coding RNAs regulate necroptosis, pyroptosis and ferroptosis, as well as their roles in cancer metastasis. However, a few important questions remain to be answered. (1) We found that there was an interaction between ferroptosis and necroptosis. As we known, iron is reported to induce oxidative stress by increasing ROS level and iron involves in various kinds of programmed cell death, such as ferroptosis and necroptosis. Iron activates ROS for GSDME-dependent pyroptosis through a Tom20–caspase–GSDME pathway in melanoma cells. Iron supplementation at a specific dosage in iron-deficient patients is effective to suppress xenograft tumor metastasis combined with clinical ROS-inducing drugs [126]. However, limited studies clarified how non-coding RNAs regulated the interactions between ferroptosis and necroptosis, ferroptosis, and pyroptosis. Thus, it is helpful to clear out the crosstalk between these different regulatory mechanisms. (2) The programmed death including ferroptosis, necroptosis and pyroptosis possessed a key role in regulation of cancer metastasis, but the role of miRNAs, lncRNAs, or circRNAs regulated programmed cell death was not clearly clarified in the metastasis of cancers. (3) It is a promising strategy in clinical cancer treatment to induce programmed cell death and identify the exact function of non-coding RNAs in the clinical therapy of cancers.

## References

[1] Jin X, Demere Z, Nair K, Ali A, Ferraro GB, Natoli T (2020). A metastasis map of human cancer cell lines. Nature.

[2] Ye X, Brabletz T, Kang Y, Longmore GD, Nieto MA, Stanger BZ (2017). Upholding a role for EMT in breast cancer metastasis. Nature.

[3] Averett C, Bhardwaj A, Arora S, Srivastava SK, Khan MA, Ahmad A (2016). Honokiol suppresses pancreatic tumor growth, metastasis and desmoplasia by interfering with tumor-stromal cross-talk. Carcinogenesis.

[4] Hoshino A, Lyden D (2017). Metastasis: lymphatic detours for cancer. Nature.

[5] Karnoub AE, Dash AB, Vo AP, Sullivan A, Brooks MW, Bell GW (2007). Mesenchymal stem cells within tumour stroma promote breast cancer metastasis. Nature.

[6] Ferguson Bennit HR, Gonda A, McMullen JRW, Kabagwira J, Wall NR (2019). Peripheral blood cell interactions of cancer-derived exosomes affect immune function. Cancer Microenviron..

[7] Galluzzi L, Kroemer G (2008). Necroptosis: a specialized pathway of programmed necrosis. Cell.

[8] Zhang T, Yin C, Boyd DF, Quarato G, Ingram JP, Shubina M (2020). Influenza virus Z-RNAs induce ZBP1-mediated necroptosis. Cell.

[9] Rickard JA, O’Donnell JA, Evans JM, Lalaoui N, Poh AR, Rogers T (2014). RIPK1 regulates RIPK3-MLKL-driven systemic inflammation and emergency hematopoiesis. Cell.

[10] Khan N, Downey J, Sanz J, Kaufmann E, Blankenhaus B, Pacis A (2020). M. tuberculosis reprograms hematopoietic stem cells to limit myelopoiesis and impair trained immunity. Cell.

[11] Mompean M, Li W, Li J, Laage S, Siemer AB, Bozkurt G (2018). The structure of the necrosome RIPK1–RIPK3 core, a human hetero-amyloid signaling complex. Cell.

[12] Dillon CP, Weinlich R, Rodriguez DA, Cripps JG, Quarato G, Gurung P (2014). RIPK1 blocks early postnatal lethality mediated by caspase-8 and RIPK3. Cell.

[13] Strilic B, Yang L, Albarran-Juarez J, Wachsmuth L, Han K, Muller UC (2016). Tumour-cell-induced endothelial cell necroptosis via death receptor 6 promotes metastasis. Nature.

[14] Dong Y, Sun Y, Huang Y, Fang X, Sun P, Dwarakanath B (2019). Depletion of MLKL inhibits invasion of radioresistant nasopharyngeal carcinoma cells by suppressing epithelial–mesenchymal transition. Ann Transl Med.

[15] Xie Y, Zhu S, Zhong M, Yang M, Sun X, Liu J (2017). Inhibition of Aurora kinase A induces necroptosis in pancreatic carcinoma. Gastroenterology.

[16] Hou J, Ju J, Zhang Z, Zhao C, Li Z, Zheng J (2019). Discovery of potent necroptosis inhibitors targeting RIPK1 kinase activity for the treatment of inflammatory disorder and cancer metastasis. Cell Death Dis.

[17] Shen F, Pan X, Li M, Chen Y, Jiang Y, He J (2020). Pharmacological inhibition of necroptosis promotes human breast cancer cell proliferation and metastasis. Onco Targets Ther.

[18] Han Q, Ma Y, Wang H, Dai Y, Chen C, Liu Y (2018). Resibufogenin suppresses colorectal cancer growth and metastasis through RIP3-mediated necroptosis. J Transl Med.

[19] Zhu F, Zhang W, Yang T, He SD (2019). Complex roles of necroptosis in cancer. J Zhejiang Univ Sci B.

[20] Deng B, Qiu B (2015). Shikonin inhibits invasiveness of osteosarcoma through MMP13 suppression. Tumour Biol.

[21] Guan YX, Zhang MZ, Chen XZ, Zhang Q, Liu SZ, Zhang YL (2018). Lnc RNA SNHG20 participated in proliferation, invasion, and migration of breast cancer cells via miR-495. J Cell Biochem.

[22] Liu XH, Sun M, Nie FQ, Ge YB, Zhang EB, Yin DD (2014). Lnc RNA HOTAIR functions as a competing endogenous RNA to regulate HER2 expression by sponging miR-331-3p in gastric cancer. Mol Cancer.

[23] Qin X, Ma D, Tan YX, Wang HY, Cai Z (2019). The role of necroptosis in cancer: a double-edged sword?. Biochim Biophys Acta Rev Cancer.

[24] Li X, Chen M, Shi Q, Zhang H, Xu S (2020). Hydrogen sulfide exposure induces apoptosis and necroptosis through lncRNA3037/miR-15a/BCL2-A20 signaling in broiler trachea. Sci Total Environ.

[25] Wang W, Shi Q, Wang S, Zhang H, Xu S (2020). Ammonia regulates chicken tracheal cell necroptosis via the LncRNA-107053293/MiR-148a-3p/FAF1 axis. J Hazard Mater.

[26] Bhattacharya S, Chalk AM, Ng AJ, Martin TJ, Zannettino AC, Purton LE (2016). Increased miR-155-5p and reduced miR-148a-3p contribute to the suppression of osteosarcoma cell death. Oncogene.

[27] Park G, Kim BS, Kim E (2020). A novel function of FAF1, which induces dopaminergic neuronal death through cell-to-cell transmission. Cell Commun Signal.

[28] Yu C, Kim BS, Kim E (2016). FAF1 mediates regulated necrosis through PARP1 activation upon oxidative stress leading to dopaminergic neurodegeneration. Cell Death Differ.

[29] Xiao H (2019). MiR-7-5p suppresses tumor metastasis of non-small cell lung cancer by targeting NOVA2. Cell Mol Biol Lett.

[30] Li M, Pan M, Wang J, You C, Zhao F, Zheng D (2020). miR-7 reduces breast cancer stem cell metastasis via inhibiting RELA to decrease ESAM expression. Mol Ther Oncolytics.

[31] Li RC, Ke S, Meng FK, Lu J, Zou XJ, He ZG (2018). CiRS-7 promotes growth and metastasis of esophageal squamous cell carcinoma via regulation of miR-7/HOXB13. Cell Death Dis.

[32] Yang L, Kong D, He M, Gong J, Nie Y, Tai S (2020). MiR-7 mediates mitochondrial impairment to trigger apoptosis and necroptosis in Rhabdomyosarcoma. Biochim Biophys Acta Mol Cell Res.

[33] Reinhart JM, Rose W, Panyard DJ, Newton MA, Liebenstein TK, Yee J (2018). RNA expression profiling in sulfamethoxazole-treated patients with a range of in vitro lymphocyte cytotoxicity phenotypes. Pharm Res Perspect.

[34] Gao SP, Sun HF, Jiang HL, Li LD, Hu X, Xu XE (2016). Loss of TIM50 suppresses proliferation and induces apoptosis in breast cancer. Tumour Biol.

[35] Zhu G, Ye J, Huang Y, Zheng W, Hua J, Yang S (2016). Receptor-interacting protein-1 promotes the growth and invasion in gastric cancer. Int J Oncol.

[36] Li X, Wang Y, Wang Y, He X (2020). MiR-141-3p ameliorates RIPK1-mediated necroptosis of intestinal epithelial cells in necrotizing enterocolitis. Aging.

[37] Li M, Huang H, Cheng F, Hu X, Liu J (2020). miR-141-3p promotes proliferation and metastasis of nasopharyngeal carcinoma by targeting NME1. Adv Med Sci.

[38] Ye Y, Li SL, Ma YY, Diao YJ, Yang L, Su MQ (2017). Exosomal miR-141-3p regulates osteoblast activity to promote the osteoblastic metastasis of prostate cancer. Oncotarget.

[39] Fang M, Huang W, Wu X, Gao Y, Ou J, Zhang X (2019). MiR-141-3p suppresses tumor growth and metastasis in papillary thyroid cancer via targeting Yin Yang 1. Anat Rec.

[40] Huang S, Wa Q, Pan J, Peng X, Ren D, Huang Y (2017). Downregulation of miR-141-3p promotes bone metastasis via activating NF-kappaB signaling in prostate cancer. J Exp Clin Cancer Res.

[41] Gu C, Hou C, Zhang S (2020). miR-425-5p improves inflammation and septic liver damage through negatively regulating the RIP1-mediated necroptosis. Inflamm Res.

[42] Hu YB, Zhang YF, Wang H, Ren RJ, Cui HL, Huang WY (2019). miR-425 deficiency promotes necroptosis and dopaminergic neurodegeneration in Parkinson’s disease. Cell Death Dis.

[43] Upton JW, Kaiser WJ, Mocarski ES (2012). DAI/ZBP1/DLM-1 complexes with RIP3 to mediate virus-induced programmed necrosis that is targeted by murine cytomegalovirus vIRA. Cell Host Microbe.

[44] Liu Y, Liu T, Lei T, Zhang D, Du S, Girani L (2019). RIP1/RIP3-regulated necroptosis as a target for multifaceted disease therapy (Review). Int J Mol Med.

[45] Yang T, Cao C, Yang J, Liu T, Lei XG, Zhang Z (2018). miR-200a-5p regulates myocardial necroptosis induced by Se deficiency via targeting RNF11. Redox Biol.

[46] Petanidis S, Domvri K, Porpodis K, Anestakis D, Freitag L, Hohenforst-Schmidt W (2020). Inhibition of kras-derived exosomes downregulates immunosuppressive BACH2/GATA-3 expression via RIP-3 dependent necroptosis and miR-146/miR-210 modulation. Biomed Pharmacother.

[47] Huang G, Xue J, Sun X, Wang J, Yu LL (2018). Necroptosis in 3-chloro-1, 2-propanediol (3-MCPD)-dipalmitate-induced acute kidney injury in vivo and its repression by miR-223-3p. Toxicology.

[48] Jiang L, Liu XQ, Ma Q, Yang Q, Gao L, Li HD (2019). hsa-miR-500a-3P alleviates kidney injury by targeting MLKL-mediated necroptosis in renal epithelial cells. FASEB J.

[49] Karshovska E, Wei Y, Subramanian P, Mohibullah R, Geissler C, Baatsch I (2020). HIF-1alpha (hypoxia-inducible factor-1alpha) promotes macrophage necroptosis by regulating miR-210 and miR-383. Arterioscler Thromb Vasc Biol.

[50] Cui Y, Yin K, Gong Y, Qu Y, Liu H, Lin H (2019). Atrazine induces necroptosis by miR-181-5p targeting inflammation and glycometabolism in carp lymphocytes. Fish Shellfish Immunol.

[51] Wang L, Shi X, Zheng S, Xu S (2020). Selenium deficiency exacerbates LPS-induced necroptosis by regulating miR-16-5p targeting PI3K in chicken tracheal tissue. Metallomics.

[52] Zhou Z, He H, Wang K, Shi X, Wang Y, Su Y (2020). Granzyme A from cytotoxic lymphocytes cleaves GSDMB to trigger pyroptosis in target cells. Science.

[53] Humphries F, Shmuel-Galia L, Ketelut-Carneiro N, Li S, Wang B, Nemmara VV (2020). Succination inactivates gasdermin D and blocks pyroptosis. Science.

[54] Wang K, Sun Q, Zhong X, Zeng M, Zeng H, Shi X (2020). Structural mechanism for GSDMD targeting by autoprocessed caspases in pyroptosis. Cell.

[55] Liu X, Zhang Z, Ruan J, Pan Y, Magupalli VG, Wu H (2016). Inflammasome-activated gasdermin D causes pyroptosis by forming membrane pores. Nature.

[56] Muendlein HI, Jetton D, Connolly WM, Eidell KP, Magri Z, Smirnova I (2020). cFLIPL protects macrophages from LPS-induced pyroptosis via inhibition of complex II formation. Science.

[57] Erkes DA, Cai W, Sanchez IM, Purwin TJ, Rogers C, Field CO (2020). Mutant BRAF and MEK inhibitors regulate the tumor immune microenvironment via pyroptosis. Cancer Discov.

[58] Wang Q, Wang Y, Ding J, Wang C, Zhou X, Gao W (2020). A bioorthogonal system reveals antitumour immune function of pyroptosis. Nature.

[59] Zhang Y, Yang H, Sun M, He T, Liu Y, Yang X (2020). Alpinumisoflavone suppresses hepatocellular carcinoma cell growth and metastasis via NLRP3 inflammasome-mediated pyroptosis. Pharm Rep.

[60] Tang Z, Ji L, Han M, Xie J, Zhong F, Zhang X (2020). Pyroptosis is involved in the inhibitory effect of FL118 on growth and metastasis in colorectal cancer. Life Sci.

[61] Yin H, Liu YG, Li F, Wang LQ, Zha JH, Xia YC (2021). Resibufogenin suppresses growth and metastasis through inducing caspase-1-dependent pyroptosis via ROS-mediated NF-kappaB suppression in non-small cell lung cancer. Anat Rec.

[62] Cheng R, Billet S, Liu C, Haldar S, Choudhury D, Tripathi M (2020). Periodontal inflammation recruits distant metastatic breast cancer cells by increasing myeloid-derived suppressor cells. Oncogene.

[63] Wu X, Mao X, Huang Y, Zhu Q, Guan J, Wu L (2020). Detection of proteins associated with the pyroptosis signaling pathway in breast cancer tissues and their significance. Int J Clin Exp Pathol.

[64] Chen Z, He M, Chen J, Li C, Zhang Q (2020). Long non-coding RNA SNHG7 inhibits NLRP3-dependent pyroptosis by targeting the miR-34a/SIRT1 axis in liver cancer. Oncol Lett.

[65] Yang F, Qin Y, Lv J, Wang Y, Che H, Chen X (2018). Silencing long non-coding RNA Kcnq1ot1 alleviates pyroptosis and fibrosis in diabetic cardiomyopathy. Cell Death Dis.

[66] Yang F, Qin Y, Wang Y, Li A, Lv J, Sun X (2018). LncRNA KCNQ1OT1 mediates pyroptosis in diabetic cardiomyopathy. Cell Physiol Biochem.

[67] Li J, Yang C, Li Y, Chen A, Li L, You Z (2018). LncRNA GAS5 suppresses ovarian cancer by inducing inflammasome formation. Biosci Rep.

[68] Liang J, Wang Q, Li JQ, Guo T, Yu D (2020). Long non-coding RNA MEG3 promotes cerebral ischemia-reperfusion injury through increasing pyroptosis by targeting miR-485/AIM2 axis. Exp Neurol.

[69] Liu Z, Wu C, Xie N, Wang P (2017). Long non-coding RNA MEG3 inhibits the proliferation and metastasis of oral squamous cell carcinoma by regulating the WNT/beta-catenin signaling pathway. Oncol Lett.

[70] Dan J, Wang J, Wang Y, Zhu M, Yang X, Peng Z (2018). LncRNA-MEG3 inhibits proliferation and metastasis by regulating miRNA-21 in gastric cancer. Biomed Pharmacother.

[71] Zhang YY, Feng HM (2017). MEG3 suppresses human pancreatic neuroendocrine tumor cells growth and metastasis by down-regulation of Mir-183. Cell Physiol Biochem.

[72] Wu L, Zhu L, Li Y, Zheng Z, Lin X, Yang C (2020). LncRNA MEG3 promotes melanoma growth, metastasis and formation through modulating miR-21/E-cadherin axis. Cancer Cell Int.

[73] Luo C, Quan Z, Zhong B, Zhang M, Zhou B, Wang S (2020). lncRNA XIST promotes glioma proliferation and metastasis through miR-133a/SOX4. Exp Ther Med.

[74] Liu X, Cui L, Hua D (2018). Long noncoding RNA XIST regulates miR-137-EZH2 axis to promote tumor metastasis in colorectal cancer. Oncol Res.

[75] Hu Y, Deng C, Zhang H, Zhang J, Peng B, Hu C (2017). Long non-coding RNA XIST promotes cell growth and metastasis through regulating miR-139-5p mediated Wnt/beta-catenin signaling pathway in bladder cancer. Oncotarget.

[76] Zhang Y, Liu J, Gao Y, Zhang Z, Zhang H. Long noncoding RNA XIST acts as a competing endogenous RNA to promote malignant melanoma growth and metastasis by sponging miR-217. Panmin Med. 2019. 10.23736/S0031-0808.19.03746-7 31833731

[77] Liu J, Yao L, Zhang M, Jiang J, Yang M, Wang Y (2019). Downregulation of LncRNA-XIST inhibited development of non-small cell lung cancer by activating miR-335/SOD2/ROS signal pathway mediated pyroptotic cell death. Aging.

[78] Zhang P, Cao L, Zhou R, Yang X, Wu M (2019). The lncRNA Neat1 promotes activation of inflammasomes in macrophages. Nat Commun.

[79] Xu X, Zhou X, Chen Z, Gao C, Zhao L, Cui Y (2020). Silencing of lncRNA XIST inhibits non-small cell lung cancer growth and promotes chemosensitivity to cisplatin. Aging.

[80] Yokoyama S, Cai Y, Murata M, Tomita T, Yoneda M, Xu L (2018). A novel pathway of LPS uptake through syndecan-1 leading to pyroptotic cell death. eLife.

[81] Song Y, Yang L, Guo R, Lu N, Shi Y, Wang X (2019). Long noncoding RNA MALAT1 promotes high glucose-induced human endothelial cells pyroptosis by affecting NLRP3 expression through competitively binding miR-22. Biochem Biophys Res Commun.

[82] Tan J, Fan J, He J, Zhao L, Tang H (2020). Knockdown of LncRNA DLX6-AS1 inhibits HK-2 cell pyroptosis via regulating miR-223-3p/NLRP3 pathway in lipopolysaccharide-induced acute kidney injury. J Bioenerg Biomembr.

[83] Wan P, Su W, Zhang Y, Li Z, Deng C, Li J (2020). LncRNA H19 initiates microglial pyroptosis and neuronal death in retinal ischemia/reperfusion injury. Cell Death Differ.

[84] Zhan JF, Huang HW, Huang C, Hu LL, Xu WW (2020). Long non-coding RNA NEAT1 regulates pyroptosis in diabetic nephropathy via mediating the miR-34c/NLRP3 axis. Kidney Blood Press Res.

[85] Li X, Zeng L, Cao C, Lu C, Lian W, Han J (2017). Long noncoding RNA MALAT1 regulates renal tubular epithelial pyroptosis by modulated miR-23c targeting of ELAVL1 in diabetic nephropathy. Exp Cell Res.

[86] Liu C, Zhuo H, Ye MY, Huang GX, Fan M, Huang XZ (2020). LncRNA MALAT1 promoted high glucose-induced pyroptosis of renal tubular epithelial cell by sponging miR-30c targeting for NLRP3. Kaohsiung J Med Sci.

[87] Wu LM, Wu SG, Chen F, Wu Q, Wu CM, Kang CM (2020). Atorvastatin inhibits pyroptosis through the lncRNA NEXN-AS1/NEXN pathway in human vascular endothelial cells. Atherosclerosis.

[88] Jin X, Jin H, Shi Y, Guo Y, Zhang H (2017). Long non-coding RNA KCNQ1OT1 promotes cataractogenesis via mir-214 and activation of the caspase-1 pathway. Cell Physiol. Biochem.

[89] Zhang Y, Liu X, Bai X, Lin Y, Li Z, Fu J, et al. Melatonin prevents endothelial cell pyroptosis via regulation of long noncoding RNA MEG3/miR-223/NLRP3 axis. J Pineal Res. 2018;64. 10.1111/jpi.12449. Epub 2017 Dec 20. 10.1111/jpi.12449 29024030

[90] Zou DM, Zhou SM, Li LH, Zhou JL, Tang ZM, Wang SH (2020). Knockdown of long noncoding RNAs of maternally expressed 3 alleviates hyperoxia-induced lung injury via inhibiting thioredoxin-interacting protein-mediated pyroptosis by binding to miR-18a. Am J Pathol.

[91] Dixon SJ, Lemberg KM, Lamprecht MR, Skouta R, Zaitsev EM, Gleason CE (2012). Ferroptosis: an iron-dependent form of nonapoptotic cell death. Cell.

[92] Liu MR, Zhu WT, Pei DS (2021). System Xc(-): a key regulatory target of ferroptosis in cancer. Investig New Drugs.

[93] Hadian K, Stockwell BR (2020). SnapShot: ferroptosis. Cell.

[94] Yang WS, SriRamaratnam R, Welsch ME, Shimada K, Skouta R, Viswanathan VS (2014). Regulation of ferroptotic cancer cell death by GPX4. Cell.

[95] Ingold I, Berndt C, Schmitt S, Doll S, Poschmann G, Buday K (2018). Selenium utilization by GPX4 is required to prevent hydroperoxide-induced ferroptosis. Cell.

[96] Wu J, Minikes AM, Gao M, Bian H, Li Y, Stockwell BR (2019). Intercellular interaction dictates cancer cell ferroptosis via NF2-YAP signalling. Nature.

[97] Hong X, Roh W, Sullivan RJ, Wong KHK, Wittner BS, Guo H (2020). The lipogenic regulator SREBF2 induces Transferrin in circulating melanoma cells and suppresses ferroptosis. Cancer Discov.

[98] Zhang D, Cui P, Dai Z, Yang B, Yao X, Liu Q (2019). Tumor microenvironment responsive FePt/MoS2 nanocomposites with chemotherapy and photothermal therapy for enhancing cancer immunotherapy. Nanoscale.

[99] Yu H, Han Z, Xu Z, An C, Xu L, Xin H (2019). RNA sequencing uncovers the key long non-coding RNAs and potential molecular mechanism contributing to XAV939-mediated inhibition of non-small cell lung cancer. Oncol Lett.

[100] Gai C, Liu C, Wu X, Yu M, Zheng J, Zhang W (2020). MT1DP loaded by folate-modified liposomes sensitizes erastin-induced ferroptosis via regulating miR-365a-3p/NRF2 axis in non-small cell lung cancer cells. Cell Death Dis.

[101] Hong YG, Xin C, Zheng H, Huang ZP, Yang Y, Zhou JD (2020). miR-365a-3p regulates ADAM10-JAK-STAT signaling to suppress the growth and metastasis of colorectal cancer cells. J Cancer.

[102] Lu J, Xu F, Lu H (2020). LncRNA PVT1 regulates ferroptosis through miR-214-mediated TFR1 and p53. Life Sci.

[103] Yang Y, Tai W, Lu N, Li T, Liu Y, Wu W (2020). lncRNA ZFAS1 promotes lung fibroblast-to-myofibroblast transition and ferroptosis via functioning as a ceRNA through miR-150-5p/SLC38A1 axis. Aging.

[104] Li C, Tian Y, Liang Y, Li Q (2020). Circ_0008035 contributes to cell proliferation and inhibits apoptosis and ferroptosis in gastric cancer via miR-599/EIF4A1 axis. Cancer Cell Int.

[105] Xian ZY, Hu B, Wang T, Cai JL, Zeng JY, Zou Q (2020). CircABCB10 silencing inhibits the cell ferroptosis and apoptosis by regulating the miR-326/CCL5 axis in rectal cancer. Neoplasma.

[106] Xu Q, Zhou L, Yang G, Meng F, Wan Y, Wang L (2020). CircIL4R facilitates the tumorigenesis and inhibits ferroptosis in hepatocellular carcinoma by regulating the miR-541-3p/GPX4 axis. Cell Biol Int.

[107] Zhang HY, Zhang BW, Zhang ZB, Deng QJ (2020). Circular RNA TTBK2 regulates cell proliferation, invasion and ferroptosis via miR-761/ITGB8 axis in glioma. Eur Rev Med Pharm Sci.

[108] Alvarez SW, Sviderskiy VO, Terzi EM, Papagiannakopoulos T, Moreira AL, Adams S (2017). NFS1 undergoes positive selection in lung tumours and protects cells from ferroptosis. Nature.

[109] Wei J, Xie Q, Liu X, Wan C, Wu W, Fang K (2020). Identification the prognostic value of glutathione peroxidases expression levels in acute myeloid leukemia. Ann Transl Med.

[110] Niu Y, Zhang J, Tong Y, Li J, Liu B (2019). Physcion 8-O-beta-glucopyranoside induced ferroptosis via regulating miR-103a-3p/GLS2 axis in gastric cancer. Life Sci.

[111] Bai T, Liang R, Zhu R, Wang W, Zhou L, Sun Y (2020). MicroRNA-214-3p enhances erastin-induced ferroptosis by targeting ATF4 in hepatoma cells. J Cell Physiol.

[112] Luo M, Wu L, Zhang K, Wang H, Zhang T, Gutierrez L (2018). miR-137 regulates ferroptosis by targeting glutamine transporter SLC1A5 in melanoma. Cell Death Differ.

[113] Xiao FJ, Zhang D, Wu Y, Jia QH, Zhang L, Li YX (2019). miRNA-17-92 protects endothelial cells from erastin-induced ferroptosis through targeting the A20–ACSL4 axis. Biochem Biophys Res Commun.

[114] Zhang K, Wu L, Zhang P, Luo M, Du J, Gao T (2018). miR-9 regulates ferroptosis by targeting glutamic-oxaloacetic transaminase GOT1 in melanoma. Mol Carcinog.

[115] Gomaa A, Peng D, Chen Z, Soutto M, Abouelezz K, Corvalan A (2019). Epigenetic regulation of AURKA by miR-4715-3p in upper gastrointestinal cancers. Sci Rep.

[116] Zhang H, Deng T, Liu R, Ning T, Yang H, Liu D (2020). CAF secreted miR-522 suppresses ferroptosis and promotes acquired chemo-resistance in gastric cancer. Mol Cancer.

[117] Xiao X, Jiang Y, Liang W, Wang Y, Cao S, Yan H (2019). miR-212-5p attenuates ferroptotic neuronal death after traumatic brain injury by targeting Ptgs2. Mol Brain.

[118] Song Y, Wang B, Zhu X, Hu J, Sun J, Xuan J (2021). Human umbilical cord blood-derived MSCs exosome attenuate myocardial injury by inhibiting ferroptosis in acute myocardial infarction mice. Cell Biol Toxicol.

[119] Tang S, Wang Y, Ma T, Lu S, Huang K, Li Q, et al. MiR-30d inhibits cardiomyocytes autophagy promoting ferroptosis after myocardial infarction. Panminerva Med. 2020 Jul 27. 10.23736/S0031-0808.20.03979-8. Online ahead of print. 10.23736/S0031-0808.20.03979-8 32720797

[120] Zhang H, He Y, Wang JX, Chen MH, Xu JJ, Jiang MH (2020). miR-30-5p-mediated ferroptosis of trophoblasts is implicated in the pathogenesis of preeclampsia. Redox Biol.

[121] Chen X, Liu G, Yuan Y, Wu G, Wang S, Yuan L (2019). NEK7 interacts with NLRP3 to modulate the pyroptosis in inflammatory bowel disease via NF-kappaB signaling. Cell Death Dis.

[122] Zhang K, Shi Z, Zhang M, Dong X, Zheng L, Li G (2020). Silencing lncRNA Lfar1 alleviates the classical activation and pyoptosis of macrophage in hepatic fibrosis. Cell Death Dis.

[123] Banoth B, Tuladhar S, Karki R, Sharma BR, Briard B, Kesavardhana S (2020). ZBP1 promotes fungi-induced inflammasome activation and pyroptosis, apoptosis, and necroptosis (PANoptosis). J Biol Chem.

[124] Shi CS, Kehrl JH (2019). Bcl-2 regulates pyroptosis and necroptosis by targeting BH3-like domains in GSDMD and MLKL. Cell Death Discov.

[125] Fritsch M, Gunther SD, Schwarzer R, Albert MC, Schorn F, Werthenbach JP (2019). Caspase-8 is the molecular switch for apoptosis, necroptosis and pyroptosis. Nature.

[126] Zhou B, Zhang JY, Liu XS, Chen HZ, Ai YL, Cheng K (2018). Tom20 senses iron-activated ROS signaling to promote melanoma cell pyroptosis. Cell Res.

[127] Wang J, Deng B, Liu Q, Huang Y, Chen W, Li J (2020). Pyroptosis and ferroptosis induced by mixed lineage kinase 3 (MLK3) signaling in cardiomyocytes are essential for myocardial fibrosis in response to pressure overload. Cell Death Dis.

[128] Zhang T, Li Y, Zhu R, Song P, Wei Y, Liang T (2019). Transcription factor p53 suppresses tumor growth by prompting pyroptosis in non-small-cell lung cancer. Oxid Med Cell Longev.

[129] Wellenstein MD, Coffelt SB, Duits DEM, van Miltenburg MH, Slagter M, de Rink I (2019). Loss of p53 triggers WNT-dependent systemic inflammation to drive breast cancer metastasis. Nature.

[130] Wang L, Zhang Z, Li M, Wang F, Jia Y, Zhang F (2019). P53-dependent induction of ferroptosis is required for artemether to alleviate carbon tetrachloride-induced liver fibrosis and hepatic stellate cell activation. IUBMB Life.

[131] Tian QQ, Xia J, Zhang X, Gao BQ, Wang W (2020). miR-331-3p inhibits tumor cell proliferation, metastasis, invasion by targeting MLLT10 in non-small cell lung cancer. Cancer Manag Res.

[132] Zhang M, Du Y, Shang J, Zhang D, Dong X, Chen H (2021). Knockdown of UCA1 restrains cell proliferation and metastasis of diffuse large B-cell lymphoma by counteracting miR-331-3p expression. Oncol Lett.

[133] Li XM, Jiao YY, Luan BH, Wu HX, Wang RR, Zhong J (2020). Long non-coding RNA MIAT promotes gastric cancer proliferation and metastasis via modulating the miR-331-3p/RAB5B pathway. Oncol Lett.

[134] Luan X, Wang Y (2018). LncRNA XLOC_006390 facilitates cervical cancer tumorigenesis and metastasis as a ceRNA against miR-331-3p and miR-338-3p. J Gynecol Oncol.

[135] Wang S, Zhang S, He Y, Huang X, Hui Y, Tang Y (2019). HOXA11-AS regulates JAK-STAT pathway by miR-15a-3p/STAT3 axis to promote the growth and metastasis in liver cancer. J Cell Biochem.

[136] Guo S, Li M, Li J, Lv Y (2020). Inhibition mechanism of lung cancer cell metastasis through targeted regulation of Smad3 by miR-15a. Oncol Lett.

[137] Fan B, Chen LP, Yuan YH, Xiao HN, Lv XS, Xia ZY (2019). MiR-15a-3p suppresses the growth and metastasis of ovarian cancer cell by targeting Twist1. Eur Rev Med Pharm Sci.

[138] Wang T, Hou J, Li Z, Zheng Z, Wei J, Song D (2017). miR-15a-3p and miR-16-1-3p negatively regulate twist1 to repress gastric cancer cell invasion and metastasis. Int J Biol Sci.

[139] Chen D, Wu D, Shao K, Ye B, Huang J, Gao Y (2017). MiR-15a-5p negatively regulates cell survival and metastasis by targeting CXCL10 in chronic myeloid leukemia. Am J Transl Res.

[140] Xu X, Zhang Y, Jasper J, Lykken E, Alexander PB, Markowitz GJ (2016). MiR-148a functions to suppress metastasis and serves as a prognostic indicator in triple-negative breast cancer. Oncotarget.

[141] Sun J, Yin A, Zhang W, Lv J, Liang Y, Li H (2020). CircUBAP2 inhibits proliferation and metastasis of clear cell renal cell carcinoma via targeting miR-148a-3p/FOXK2 pathway. Cell Transplant.

[142] Cai Q, Zhu A, Gong L (2018). Exosomes of glioma cells deliver miR-148a to promote proliferation and metastasis of glioblastoma via targeting CADM1. Bull Cancer.

[143] Tang T, Yang Z, Zhu Q, Wu Y, Sun K, Alahdal M (2018). Up-regulation of miR-210 induced by a hypoxic microenvironment promotes breast cancer stem cells metastasis, proliferation, and self-renewal by targeting E-cadherin. Faseb J.

[144] Yang X, Shi L, Yi C, Yang Y, Chang L, Song D (2017). MiR-210-3p inhibits the tumor growth and metastasis of bladder cancer via targeting fibroblast growth factor receptor-like 1. Am J Cancer Res.

[145] Petrozza V, Costantini M, Tito C, Giammusso LM, Sorrentino V, Cacciotti J (2020). Emerging role of secreted miR-210-3p as potential biomarker for clear cell renal cell carcinoma metastasis. Cancer Biomark.

[146] Ji J, Rong Y, Luo CL, Li S, Jiang X, Weng H (2018). Up-regulation of hsa-miR-210 promotes venous metastasis and predicts poor prognosis in hepatocellular carcinoma. Front Oncol.

[147] Qu A, Du L, Yang Y, Liu H, Li J, Wang L (2014). Hypoxia-inducible MiR-210 is an independent prognostic factor and contributes to metastasis in colorectal cancer. PLoS ONE.

[148] Roth E, Cao J (2015). miR-181 suppresses metastasis via MMP-14. Aging.

[149] Yang CC, Hung PS, Wang PW, Liu CJ, Chu TH, Cheng HW (2011). miR-181 as a putative biomarker for lymph-node metastasis of oral squamous cell carcinoma. J Oral Pathol Med.

[150] Zhang H, Yang K, Ren T, Huang Y, Tang X, Guo W (2018). miR-16-5p inhibits chordoma cell proliferation, invasion and metastasis by targeting Smad3. Cell Death Dis.

[151] Xu XP, Peng XQ, Yin XM, Liu Y, Shi ZY (2020). miR-34a-5p suppresses the invasion and metastasis of liver cancer by targeting the transcription factor YY1 to mediate MYCT1 upregulation. Acta Histochem.

[152] Krzeszinski JY, Wei W, Huynh H, Jin Z, Wang X, Chang TC (2019). Author Correction: miR-34a blocks osteoporosis and bone metastasis by inhibiting osteoclastogenesis and Tgif2. Nature.

[153] Li J, Lam M (2015). Registered report: the microRNA miR-34a inhibits prostate cancer stem cells and metastasis by directly repressing CD44. eLife.

[154] Yan X, Tang B, Chen B, Shan Y, Yang H (2019). Replication study: the microRNA miR-34a inhibits prostate cancer stem cells and metastasis by directly repressing CD44. eLife.

[155] Zhang Y, Song Z, Li X, Xu S, Zhou S, Jin X (2020). Long noncoding RNA KCNQ1OT1 induces pyroptosis in diabetic corneal endothelial keratopathy. Am J Physiol Cell Physiol.

[156] Zhang C, Gong Y, Li N, Liu X, Zhang Y, Ye F (2021). Long non-coding RNA Kcnq1ot1 promotes sC5b-9-induced podocyte pyroptosis by inhibiting miR-486a-3p and upregulating NLRP3. Am J Physiol Cell Physiol.

[157] Xie C, Wu W, Tang A, Luo N, Tan Y (2019). lncRNA GAS5/miR-452-5p reduces oxidative stress and pyroptosis of high-glucose-stimulated renal tubular cells. Diabetes Metab Syndr Obes.

[158] Xu Y, Fang H, Xu Q, Xu C, Yang L, Huang C (2020). LncRNA GAS5 inhibits NLRP3 inflammasome activation-mediated pyroptosis in diabetic cardiomyopathy by targeting miR-34b-3p/AHR. Cell Cycle.

[159] Li J, Zi Y, Wang W, Li Y (2018). Long noncoding RNA MEG3 inhibits cell proliferation and metastasis in chronic myeloid leukemia via targeting miR-184. Oncol. Res.

[160] Hu Y, Deng C, Zhang H, Zhang J, Peng B, Hu C (2017). Long non-coding RNA XIST promotes cell growth and metastasis through regulating miR-139-5p mediated Wnt/β-catenin signaling pathway in bladder cancer. Oncotarget.

[161] Li S, Hao J, Hong Y, Mai J, Huang W (2020). Long non-coding RNA NEAT1 promotes the proliferation, migration, and metastasis of human breast-cancer cells by inhibiting miR-146b-5p expression. Cancer Manag Res.

[162] Zou JX, Ge TW (2020). Long non-coding RNA NEAT1 promotes tumor development and metastasis through targeting miR-224-5p in malignant melanoma. Eur Rev Med Pharm Sci.

[163] Liu Y, Wang Y, Fu X, Lu Z (2018). Long non-coding RNA NEAT1 promoted ovarian cancer cells’ metastasis through regulation of miR-382-3p/ROCK1 axial. Cancer Sci.

[164] Sun Z, Zhang T, Chen B (2019). Long Non-coding RNA metastasis-associated lung adenocarcinoma transcript 1 (MALAT1) promotes proliferation and metastasis of osteosarcoma cells by targeting c-Met and SOX4 via miR-34a/c-5p and miR-449a/b. Med Sci Monit.

[165] Zhao Z, Liang S, Sun F (2020). LncRNA DLX6-AS1 promotes malignant phenotype and lymph node metastasis in prostate cancer by inducing LARGE methylation. Front Oncol.

[166] Zhang N, Meng X, Mei L, Zhao C, Chen W. LncRNA DLX6-AS1 promotes tumor proliferation and metastasis in osteosarcoma through modulating miR-641/HOXA9 signaling pathway. J Cell Biochem. 2019 Mar 6.10.1002/jcb.28426. Online ahead of print. 10.1002/jcb.28426 30838699

[167] Wu SB, Wang HQ (2020). Upregulation of long noncoding RNA DLX6-AS1 promotes cell growth and metastasis in esophageal squamous cell carcinoma via targeting miR-577. Eur Rev Med Pharm Sci.

[168] Zhao J, Liu HR (2019). Down-regulation of long noncoding RNA DLX6-AS1 defines good prognosis and inhibits proliferation and metastasis in human epithelial ovarian cancer cells via Notch signaling pathway. Eur Rev Med Pharm Sci.

[169] Zhang T, Lei F, Jiang T, Xie L, Huang P, Li P (2019). H19/miR-675-5p targeting SFN enhances the invasion and metastasis of nasalpharyngeal cancer cells. Curr Mol Pharmacol.

[170] Si H, Chen P, Li H, Wang X (2019). Long non-coding RNA H19 regulates cell growth and metastasis via miR-138 in breast cancer. Am J Transl Res.

[171] Lv M, Zhong Z, Huang M, Tian Q, Jiang R, Chen J (2017). lncRNA H19 regulates epithelial–mesenchymal transition and metastasis of bladder cancer by miR-29b-3p as competing endogenous RNA. Biochim Biophys Acta Mol Cell Res.

[172] Xu Y, Lin J, Jin Y, Chen M, Zheng H, Feng J (2019). The miRNA hsa-miR-6515-3p potentially contributes to lncRNA H19-mediated-lung cancer metastasis. J Cell Biochem.

[173] Qiu F, Zhang MR, Zhou Z, Pu JX, Zhao XJ (2019). lncRNA MIR503HG functioned as a tumor suppressor and inhibited cell proliferation, metastasis and epithelial–mesenchymal transition in bladder cancer. J. Cell Biochem.

[174] Wang H, Liang L, Dong Q, Huan L, He J, Li B (2018). Long noncoding RNA miR503HG, a prognostic indicator, inhibits tumor metastasis by regulating the HNRNPA2B1/NF-κB pathway in hepatocellular carcinoma. Theranostics.

[175] Qi G, Li L (2020). Long non-coding RNA PVT1 contributes to cell growth and metastasis in non-small-cell lung cancer by regulating miR-361-3p/SOX9 axis and activating Wnt/β-catenin signaling pathway. Biomed Pharmacother.

[176] Wang Z, Su M, Xiang B, Zhao K, Qin B (2019). Circular RNA PVT1 promotes metastasis via miR-145 sponging in CRC. Biochem Biophys Res Commun.

[177] Yan M, Gao H, Lv Z, Liu Y, Zhao S, Gong W (2020). Circular RNA PVT1 promotes metastasis via regulating of miR-526b/FOXC2 signals in OS cells. J Cell Mol Med.

[178] Yan M, Pan XF, Liu Y, Zhao S, Gong WQ, Liu W (2020). Long noncoding RNA PVT1 promotes metastasis via miR-484 sponging in osteosarcoma cells. Eur Rev Med Pharm Sci.

[179] Zhu X, Du J, Gu Z (2020). Circ-PVT1/miR-106a-5p/HK2 axis regulates cell growth, metastasis and glycolytic metabolism of oral squamous cell carcinoma. Mol Cell Biochem.

[180] Niu J, Song X, Zhang X (2020). Regulation of lncRNA PVT1 on miR-125 in metastasis of gastric cancer cells. Oncol Lett.

[181] Lan T, Yan X, Li Z, Xu X, Mao Q, Ma W (2017). Long non-coding RNA PVT1 serves as a competing endogenous RNA for miR-186-5p to promote the tumorigenesis and metastasis of hepatocellular carcinoma. Tumour Biol.

[182] Pan Y, Liu L, Cheng Y, Yu J, Feng Y (2020). Amplified LncRNA PVT1 promotes lung cancer proliferation and metastasis by facilitating VEGFC expression. Biochem Cell Biol=Biochim Biol Cell.

[183] Liu B, Tan Z, Jiang Y, Chen Y, Chen Y, Ling K (2018). Correlation between the expression of miR150 and FOXO4 and the local recurrence and metastasis of nasopharyngeal carcinoma after intensive radiotherapy. J BUON.

[184] Ba Y, Liu Y, Li C, Zhu Y, Xing W (2020). HIPK3 promotes growth and metastasis of esophageal squamous cell carcinoma via regulation of miR-599/c-MYC axis. Onco Targets Ther.

[185] Ji W, Wang Q, Yang J (2020). LncRNA HOXD-AS1 promotes the metastasis of human hepatocellular carcinoma via modulating miR-326/SLC27A4. Cancer Cell Int.

[186] Li Y, Gao Y, Xu Y, Ma H, Yang M (2015). Down-regulation of miR-326 is associated with poor prognosis and promotes growth and metastasis by targeting FSCN1 in gastric cancer. Growth Factors.

[187] Liu W, Zhang B, Xu N, Wang MJ, Liu Q (2017). miR-326 regulates EMT and metastasis of endometrial cancer through targeting TWIST1. Eur Rev Med Pharm Sci.

[188] Valencia K, Martín-Fernández M, Zandueta C, Ormazábal C, Martínez-Canarias S, Bandrés E (2013). miR-326 associates with biochemical markers of bone turnover in lung cancer bone metastasis. Bone.

[189] Xia YH, Ren L, Li JZ, Gao F (2019). Role of miR-541-3p/TMPRSS4 in the metastasis and EMT of hepatocellular carcinoma. Eur Rev Med Pharm Sci.

[190] Yan A, Yang C, Chen Z, Li C, Cai L (2015). MiR-761 promotes progression and metastasis of non-small cell lung cancer by targeting ING4 and TIMP2. Cell Physiol Biochem.

[191] Li C, Zhou H (2020). Circular RNA hsa_circRNA_102209 promotes the growth and metastasis of colorectal cancer through miR-761-mediated Ras and Rab interactor 1 signaling. Cancer Med.

[192] Zhang S, Cai J, Xie W, Luo H, Yang F (2018). miR-202 suppresses prostate cancer growth and metastasis by targeting PIK3CA. Exp Ther Med.

[193] Zhang J, Piao CD, Ding J, Li ZW (2020). LncRNA MALAT1 facilitates lung metastasis of osteosarcomas through miR-202 sponging. Sci Rep.

[194] Niu Y, Zhang J, Tong Y, Li J, Liu B (2019). Physcion 8-O-β-glucopyranoside induced ferroptosis via regulating miR-103a-3p/GLS2 axis in gastric cancer. Life Sci.

[195] Zheng J, Liu Y, Qiao Y, Zhang L, Lu S. miR-103 promotes proliferation and metastasis by targeting KLF4 in gastric cancer. Int J Mol Sci. 2017;18:910. 10.3390/ijms18050910. 10.3390/ijms18050910 PMC5454823 28445396

[196] Han LL, Yin XR, Zhang SQ (2018). miR-103 promotes the metastasis and EMT of hepatocellular carcinoma by directly inhibiting LATS2. Int J Oncol.

[197] Chen HY, Lin YM, Chung HC, Lang YD, Lin CJ, Huang J (2012). miR-103/107 promote metastasis of colorectal cancer by targeting the metastasis suppressors DAPK and KLF4. Cancer Res.

[198] Plum PS, Warnecke-Eberz U, Drebber U, Chon SH, Alakus H, Hölscher AH (2019). Upregulation of miR-17-92 cluster is associated with progression and lymph node metastasis in oesophageal adenocarcinoma. Sci Rep.

[199] Bahari F, Emadi-Baygi M, Nikpour P (2015). miR-17-92 host gene, uderexpressed in gastric cancer and its expression was negatively correlated with the metastasis. Indian J Cancer.

[200] Yang J, Qiu Q, Qian X, Yi J, Jiao Y, Yu M (2019). Long noncoding RNA LCAT1 functions as a ceRNA to regulate RAC1 function by sponging miR-4715-5p in lung cancer. Mol Cancer.

[201] Zhang T, Hu Y, Ju J, Hou L, Li Z, Xiao D (2016). Downregulation of miR-522 suppresses proliferation and metastasis of non-small cell lung cancer cells by directly targeting DENN/MADD domain containing 2D. Sci Rep.

[202] Du F, Li Z, Zhang G, Shaoyan S, Geng D, Tao Z (2020). SIRT2, a direct target of miR-212-5p, suppresses the proliferation and metastasis of colorectal cancer cells. J Cell Mol Med.

[203] Zhou C, Tan DM, Chen L, Xu XY, Sun CC, Zong LJ (2017). Effect of miR-212 targeting TCF7L2 on the proliferation and metastasis of cervical cancer. Eur Rev Med Pharm Sci.

[204] Ma F, Li W, Liu C, Li W, Yu H, Lei B (2017). MiR-23a promotes TGF-β1-induced EMT and tumor metastasis in breast cancer cells by directly targeting CDH1 and activating Wnt/β-catenin signaling. Oncotarget.

[205] Bao L, You B, Shi S, Shan Y, Zhang Q, Yue H (2018). Metastasis-associated miR-23a from nasopharyngeal carcinoma-derived exosomes mediates angiogenesis by repressing a novel target gene TSGA10. Oncogene.

[206] Li X, Liu X, Xu W, Zhou P, Gao P, Jiang S (2013). c-MYC-regulated miR-23a/24-2/27a cluster promotes mammary carcinoma cell invasion and hepatic metastasis by targeting Sprouty2. J Biol Chem.

[207] Guo W, Wang H, Yang Y, Guo S, Zhang W, Liu Y (2017). Down-regulated miR-23a contributes to the metastasis of cutaneous melanoma by promoting autophagy. Theranostics.

[208] Gao Y, Luo X, Zhang J (2020). Sp1-mediated up-regulation of lnc00152 promotes invasion and metastasis of retinoblastoma cells via the miR-30d/SOX9/ZEB2 pathway. Cell Oncol.

