# Correction: Non-coding RNAs in necroptosis, pyroptosis and ferroptosis in cancer metastasis

**DOI:** 10.1038/s41420-021-00617-7

**Published:** 2021-08-31

**Authors:** Yan Liu, Qiuyun Chen, Yanan Zhu, Tiying Wang, Lijuan Ye, Hei Han, Zhihong Yao, Zuozhang Yang

**Affiliations:** 1 Bone and Soft Tissue Tumors Research Center of Yunnan Province, Department of Orthopaedics, The Third Affiliated Hospital of Kunming Medical University (Cancer Hospital of Yunnan Province), Kunming, Yunnan China; 2 Department of Pathology, The Third Affiliated Hospital of Kunming Medical University (Cancer Hospital of Yunnan Province), Kunming, Yunnan China

**Keywords:** Cancer, Mechanisms of disease

Correction to: *Cell Death Discovery* 10.1038/s41420-021-00596-9, published online 11 August 2021

The original version of this article unfortunately contained a mistake in Lei Han’s name. We apologize for the error. The original article has been corrected.

